# Modelling Odor Decoding in the Antennal Lobe by Combining Sequential Firing Rate Models with Bayesian Inference

**DOI:** 10.1371/journal.pcbi.1004528

**Published:** 2015-10-09

**Authors:** Dario Cuevas Rivera, Sebastian Bitzer, Stefan J. Kiebel

**Affiliations:** 1 Department of Psychology, Technische Universität, Dresden, Germany; 2 Biomagnetic Centre, Department of Neurology, University Hospital Jena, Jena, Germany; 3 Department of Neurology, Max Planck Institute for Human Cognitive and Brain Sciences, Leipzig, Germany; Northwestern University, UNITED STATES

## Abstract

The olfactory information that is received by the insect brain is encoded in the form of spatiotemporal patterns in the projection neurons of the antennal lobe. These dense and overlapping patterns are transformed into a sparse code in Kenyon cells in the mushroom body. Although it is clear that this sparse code is the basis for rapid categorization of odors, it is yet unclear how the sparse code in Kenyon cells is computed and what information it represents. Here we show that this computation can be modeled by sequential firing rate patterns using Lotka-Volterra equations and Bayesian online inference. This new model can be understood as an ‘intelligent coincidence detector’, which robustly and dynamically encodes the presence of specific odor features. We found that the model is able to qualitatively reproduce experimentally observed activity in both the projection neurons and the Kenyon cells. In particular, the model explains mechanistically how sparse activity in the Kenyon cells arises from the dense code in the projection neurons. The odor classification performance of the model proved to be robust against noise and time jitter in the observed input sequences. As in recent experimental results, we found that recognition of an odor happened very early during stimulus presentation in the model. Critically, by using the model, we found surprising but simple computational explanations for several experimental phenomena.

## Author Summary

Odor recognition in the insect brain is amazingly fast but still not fully understood. It is known that recognition is performed in three stages. In the first stage, the sensors respond to an odor by displaying a reproducible neuronal pattern. This code is turned, in the second and third stages, into a sparse code, that is, only relatively few neurons activate over hundreds of milliseconds. It is generally assumed that the insect brain uses this temporal code to recognize an odor. We propose a new model of how this temporal code emerges using sequential activation of groups of neurons. We show that these sequential activations underlie a fast and accurate recognition which is highly robust against neuronal or sensory noise. This model replicates several key experimental findings and explains how the insect brain achieves both speed and robustness of odor recognition as observed in experiments.

## Introduction

Understanding how a brain encodes and decodes olfactory input has been an active field of study for decades [1,2]. The relatively simple circuitry in the insect brain for odor processing offers a good opportunity to understand the basic principles of sensory processing in brains. Some findings have been key in understanding how the insect brain makes sense of the olfactory information that it acquires from the outside world: (i) There are three stages of stimulus processing: in the antennae, the receptor neurons bond with odorants creating a time-invariant spatial pattern of activations in these neurons, which is sent to the antennal lobe [3]. In the antennal lobe, the projection neurons (PNs) react with odor-specific spatiotemporal patterns [4], whose duration far surpasses that of the stimulus itself [5]. In the mushroom body (MB), the target of the PNs, a small number of highly-specific Kenyon cells (KC) respond with short-lived activation periods, often only with a single spike. (ii) Odor-specific trajectories can be measured in the PN firing rate phase space, and the separation between the trajectories for different odors is greatest during a period of slow dynamics which lasts for about 1.5s after odor onset. (iii) The spatiotemporal patterns that arise in the PN population encode the identity of the odor [6], but can be difficult to differentiate for any two odors [7]. It is only at the KC level that the trajectories are easily identifiable, due to the sparseness of KC responses [2].

In response to an odor, only a few of KCs fire spikes (population sparseness) and the firing rates are limited to usually one or two spikes during the presentation of the odor (lifetime sparseness). The causes of this KC sparseness and its precise role in odor decoding are still unknown. It has been suggested that the KCs act as coincidence detectors [5,8], i.e., a KC becomes active only when a number of its input PNs are active. Another proposal offers an explanation for the lifetime sparseness of the response based on spike frequency adaptation [9], albeit without providing an explicit functional role for the sparseness.

During the period of slow dynamics in the response of the PNs to a stimulus, the firing rates of single PNs rise and fall sequentially in an odor-specific order, creating a sequence of “active” PNs through time [10]. Such a sequence-generating device could be the basis of an odor recognition mechanism at the KC or downstream levels. There are two previous modelling approaches addressing this. The first approach used Lotka-Volterra equations to model PN activations [11]. The solution of these equations visits the vicinity of a set of equilibrium points, giving rise to a trajectory in the phase space akin to those observed in experiments. This model is an abstract system that behaves in a similar way to that observed in the PN population, albeit without providing a mechanism for decoding stimulus responses at the KC level. The second approach presented a computational mechanism through which lifetime sparseness can be achieved in the KC population as information is passed on from the PNs [9]; however, the sequential nature of the activity at both the PN and KC level was not part of this model.

Although these two previous computational models addressed fundamental questions, it is still unclear how the PNs and KCs interact mechanistically to enable odor recognition. In particular, the following two key questions remain to be answered: (i) How does the insect brain achieve its speed and robustness of odor recognition by transforming highly overlapping spatiotemporal PN patterns into a potentially unique and easily recognizable sequence of KC activations? (ii) What is the mechanism behind the hypothesized coincidence detection of the KCs?

In this work, we present a new model that addresses these two questions. The model combines a nonlinear generative model with approximate Bayesian inference for nonlinear dynamical systems [12–14]. Specifically, as a generative model, we use a modified version of the Laurent-Rabinovich model introduced in [11], which is based on sequential neuronal dynamics to describe the dynamics of PNs. In the proposed generative model, the PN activity is the input to the model. We propose to use Bayesian inference to infer the states of the hidden variables, i.e. the firing rates of the KCs. The model dynamics exhibit the observed behavior of both PNs and KCs in the insect brain and mechanistically explains how sparse code in the KCs emerges from the dense coding of the PNs. In addition, the Bayesian inference approach enables the recognition of odors from PN activation dynamics, which may be understood as an ‘intelligent coincidence detector’ implemented by the KCs. In sum, we present a model which (1) replicates experimentally observed dynamics at two hierarchical levels of the odor recognition system of insects, (2) provides simple explanations for several key experimental findings and (3) implements fast and robust odor recognition based on firing rate input.

## Results

We developed a model of how the insect brain encodes the qualities of an odor and subsequently decodes this information to identify the odor. To do this, we assumed that the underlying encoding mechanism in the brain is based on sequential neuronal dynamics [15], as in the Laurent-Rabinovich model introduced in [11]. The proposed model consists of two components. The first is a generative model in accordance with key experimental findings that will be briefly reviewed below. The second is a Bayesian inference scheme based on this generative model. As we will show, this Bayesian model can recognize synthetic PN input using sequential KC activity. Note that the Bayesian inference scheme is the key device to derive a fast and robust decoding model while maintaining, at a firing rate level, neurobiological plausibility by relying on the Bayesian brain hypothesis [12,16–19].

Based on experimental findings that the representations for different odors appear to be well differentiated at the KC level (as opposed to the PN level) [20], we refer to successful odor recognition when the model can successfully reproduce the spatiotemporal KC representation expected for a specific odor (see Methods for details).

In the following, we first briefly review the experimental findings which we used to derive the generative model for the activity of the KC and PN population, followed by the model description and results.

### Previous experimental findings

#### Slow evolution of PN firing pattern

In response to a stimulus, the PNs exhibit temporal spiking patterns in two frequency ranges that are independent of the frequencies of variation in the stimulus [4]: in fast, synchronized oscillations of 20–30Hz [4] and in a slower evolution, on a timescale of seconds. The slow evolution of the response to an odor is divided into three phases [7]: the onset sequential phase, which occurs first, is characterized by sequential dynamics, in which the whole PN population responds with spatial patterns of activation which evolve in time. When the stimulus is presented for more than ca. 1.5s, the system enters the steady state phase, where it exhibits steady state dynamics, with stagnant PN activity; when the stimulus lasts less than 1.5s, this second phase is skipped. The steady state phase lasts until stimulus offset, which triggers the offset sequential phase, where again sequential dynamics are observed, different from those in the first phase. The activity then slowly fades back to baseline activity over the course of a few seconds.

#### Sequential firing patterns of PNs

Single PNs can respond to a stimulus in a multiphasic way [4]: they can be at a baseline firing rate of around 3Hz [5], in an excited state or inhibited under baseline. Correspondingly, the PN population can be divided into three groups at any given time during the response: inhibited, excited and at baseline. The ensuing response can be seen as a sequence of spatial patterns of activation, each being a combination of excited, inhibited and baseline PNs. The duration of each of these patterns is not fixed, even for the same insect-odor combination, but the order in which they appear (i.e. the order of the sequence) is reproducible across trials [7,10]. The representation of an odor in the PNs of the antennal lobe can be understood as a sequence whose elements (the spatial patterns) appear in the same order for different trials of the same odor, even if the exact timing changes. These elements are largely overlapping: a PN that is excited (or inhibited) during one of these elements will probably also be excited (or inhibited) during other elements of the sequence for that odor or for other odors.

The evolution of these sequences of spatial patterns can be understood as trajectories in the phase space of the firing rates of the PNs. While all trajectories are odor-specific throughout their duration, the maximum separation between trajectories for different odors appears during the first phase of the response [7].

#### Sparse KC code

The dense and largely overlapping spatial patterns in the PNs are transformed into sparse, highly odor-specific sequences of spikes produced by a small number of odor-specific KCs, while all other KCs remain silent. This means that the KC population reacts to a stimulus in clusters comprising a small number of KCs that increase their firing rate, as suggested for example in [3] and can be seen in the raster plots in [5]. Furthermore, it has been found that, while the number of odors that a KC responds to is much lower than that of the PNs, some KCs activate in response to more than one odor [5].

### Previous modeling work for sequential activity

Previous efforts for modelling the sequential dynamics during the onset sequential phase of an odor response in the antennal lobe used the Lotka-Volterra equations [11,21]. This model used the Lotka-Volterra equations to obtain reproducible sequential dynamics in the PN population. The equations are the following:

$${\dot{x}}_{i}={\text{x}}_{i}\left(\sigma_{i}(I)+\sum_{i\neq j}\rho_{ij}(I)x_{j}\right)+\eta_{i}$$ 1

where x_i_ is the firing rate of the i-th neuron, *σ*_*i*_ is a parameter, *η* is noise and *ρ*_*ij*_ is the connectivity matrix among the neurons; *I* is the input to the system, i.e. the odor being perceived. Because of the continuum of possible input odors, the parameters *ρ* and *σ* are continuous functions of the input *I*. Under the conditions over *ρ*_*ij*_ and *σ*_*i*_ given in [22] the system has a set of equilibrium points *Q*_*i*_ = (0,0,…,*σ*_*i*_,…,0), where the non-zero entry is at the i-th position, and its solution presents a stable heteroclinic sequence (SHS), which is the union of these equilibrium points and trajectories that join them in a specific sequence. An odor is represented by a sequence of equilibrium points visited by the solution. When presented with a stimulus, which sets a value for *σ*_*i*_ and the connectivity matrix *ρ*_*ij*_, the system responds by visiting a sequence of equilibrium points in which the order of the points is constant across trials.

Although this model (which we call the Laurent-Rabinovich model from now on) captures the most prominent feature of the PN data, i.e., neuronal sequential activations, the model has several limitations. Firstly, there is no mechanism for how the sensory input is received by the model. This means that the model cannot recognize one specific odor among many possible alternatives but rather follows the dynamics of a specific, pre-set odor. Secondly, as the input is only used to fix the model parameters, there is no way of finding out how robust the model is against sensory or neuronal noise. For example, neuronal noise may mean that for a given odor a PN does not fire although it should, or if a PN does fire although it should not. Clearly, a model of odor recognition should be robust against such neuronal noise. Thirdly, the Laurent-Rabinovich model is meant to model PN activity but does not describe the sparse KC activity. This means that one of the most prominent questions, i.e. how dense PN activity is transformed into the sparse KC code, cannot be addressed by the model proposed in [11].

### A novel model of odor recognition

In this paper, we build on the core idea of the Laurent-Rabinovich model that odor recognition is based on neuronal sequences and extend the model in three ways.

Firstly, experimental results show that KCs respond to more than one odor and are activated in small groups [5,20]. Motivated by these results, we replace the rather simple neuronal sequences of Eq 1 used in [11] by sequences of small neuronal clusters and use these equations to describe sequences in KCs as opposed to the PNs. As we will show below, this extension massively increases the number of possible odors that can be recognized by the model and is critical in explaining how a KC can represent multiple odors. In addition, this cluster extension makes the decoding at the KC level highly robust against failures of single KCs because at each point in time during odor recognition multiple KCs sparsely share the decoding.

Secondly, we combine the KC-cluster sequences with a model for PN activity. This will enable us to model the hierarchical decoding of the dense PN code by sparse KC activity.

Thirdly, we combine the resulting PC/KN model with Bayesian inference. This will make the model a recognition model, i.e. the model can receive and decode PN input. Critically, we will show that this recognition model can identify specific odors (out of a selection of odors) very rapidly, and is robust against several noise sources, for instance unexpectedly activated/inactivated PNs.

In sum, these three extensions enable us to answer our two questions: (i) How does the insect brain achieve its speed and robustness of odor recognition by transforming highly-overlapping spatiotemporal PN patterns into a potentially unique and easily recognizable sequence of KC activations? (ii) What is the mechanism underlying the hypothesized coincidence detection of the KCs?

In the following we will describe each of the three extensions in detail.

#### Sequences of KC clusters

**Fig 1:**
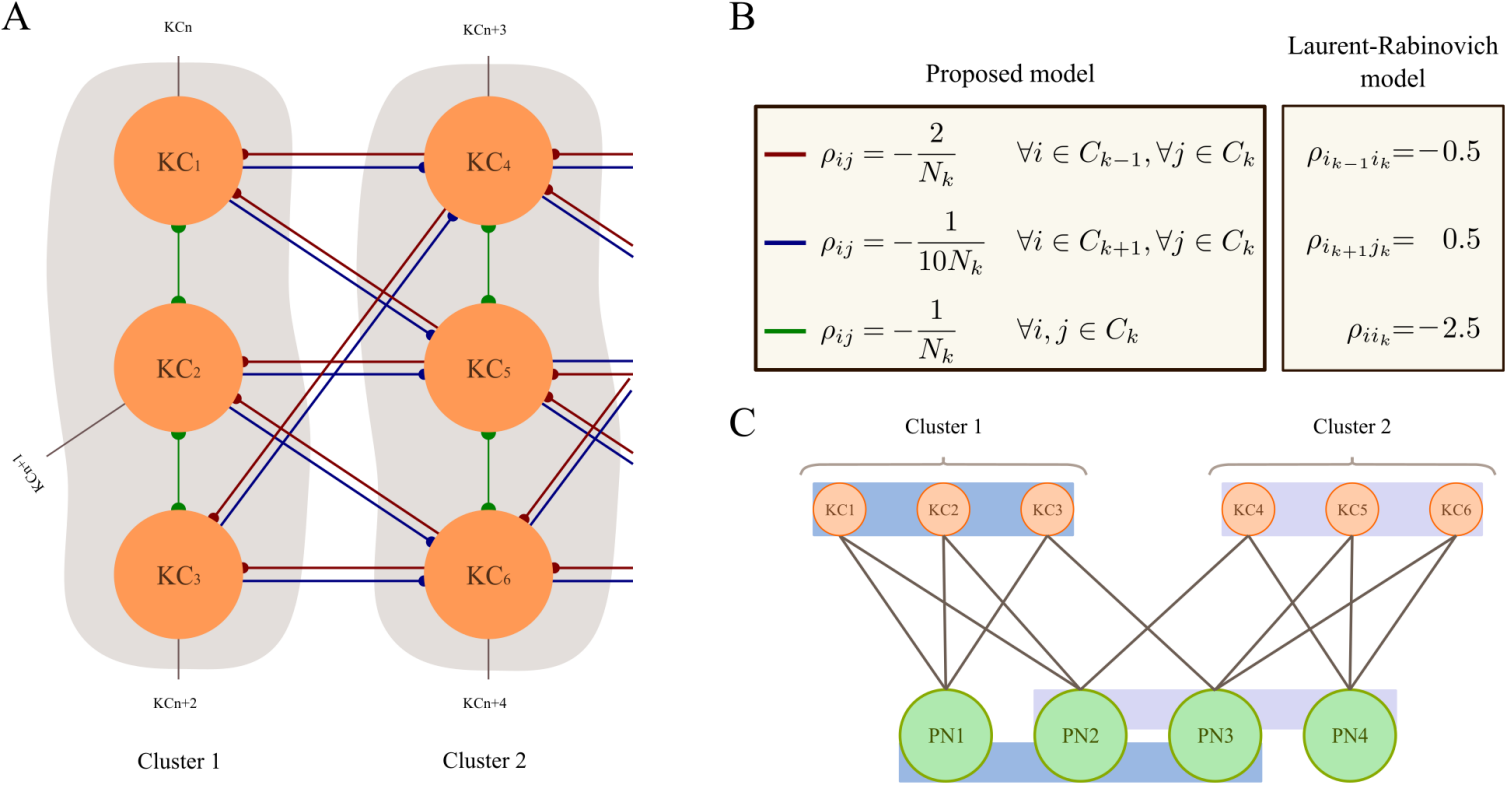
Connectivity among KCs and PNs. (A) Clusters of KCs and their connectivity. The connections shown here are the low-inhibition connections (blue) and high-inhibition (red) between specific KCs and the medium inhibition between members of a cluster (green). The high-inhibition all-to-all connections are not depicted. Each neuron is connected (as an example) to three other neurons outside of its cluster. A line with a single dot at the end means that the connection is one-way only. The neurons in one cluster connect to each other and to elements outside the cluster. Those neurons that receive input from more than one KC in a cluster form the cluster to be activated next. Each KC could connect with a neuron that is not part of the next cluster; these connections are labeled KCn, KCn+1, etc. and arise because KCs can belong to other clusters as well. (B) Conditions for the connectivity matrix in the proposed model and in the [11] model. In the proposed model, all components of the connectivity matrix ρ are negative (inhibition), whereas the [11] model uses both inhibition and excitation. *C*_*k*_ is the k-th cluster, i.e. *i* ϵ *C*_*k*_ for all neurons in cluster k. (C) Example of connections between KCs and PNs. KCs work as coincidence detectors, being activated only when both the PNs they are connected to are activated. Shown, a population of 6 KCs divided in two clusters. Four PNs are connected to the KCs so that each KC sees half of the PN population. When PNs 1, 2 and 3 are active, cluster 1 is activated. Cluster 2 is activated with PNs 2, 3 and 4.

Because KCs respond to more than one odor and are activated in small groups [5,20], we modified the conditions on the connectivity matrix presented in [11,21,22] to allow clusters of KCs, instead of single neurons, to represent an element in an odor-specific sequence (see Fig 1). With this neuronal cluster approach, a single KC can be part of the representations of multiple odors and activate more than once in response to a single odor. This enables us to encode several sequences (odors) within a single connectivity matrix, while a single KC can respond to more than one odor. This was not possible in the Laurent-Rabinovich model.

Inhibitory connections between the KCs, which translate into negative constants *ρ*_*ij*_, create the sequential dynamics between neuronal clusters. We used two general types of inhibitory connections with high and low levels of inhibition between different KCs. Connections with low inhibition encode the neuronal sequences associated with odors. They only exist between two neurons if they appear in the same sequence. Connections with high inhibition exist between two neurons that do not appear together in any sequence. We implement this high inhibition, which is almost all-to-all, as direct connections between KCs, but it can equivalently be implemented as a pool of inhibition that is connected to all neurons in the population. Note that there is empirical evidence for the latter connection scheme [23] supporting our model assumption that the effect of KC activity on other KCs is mostly inhibitory. A full description of these connections can be found in the section ‘Model parameters used for simulations’ below.

**Fig 2:**
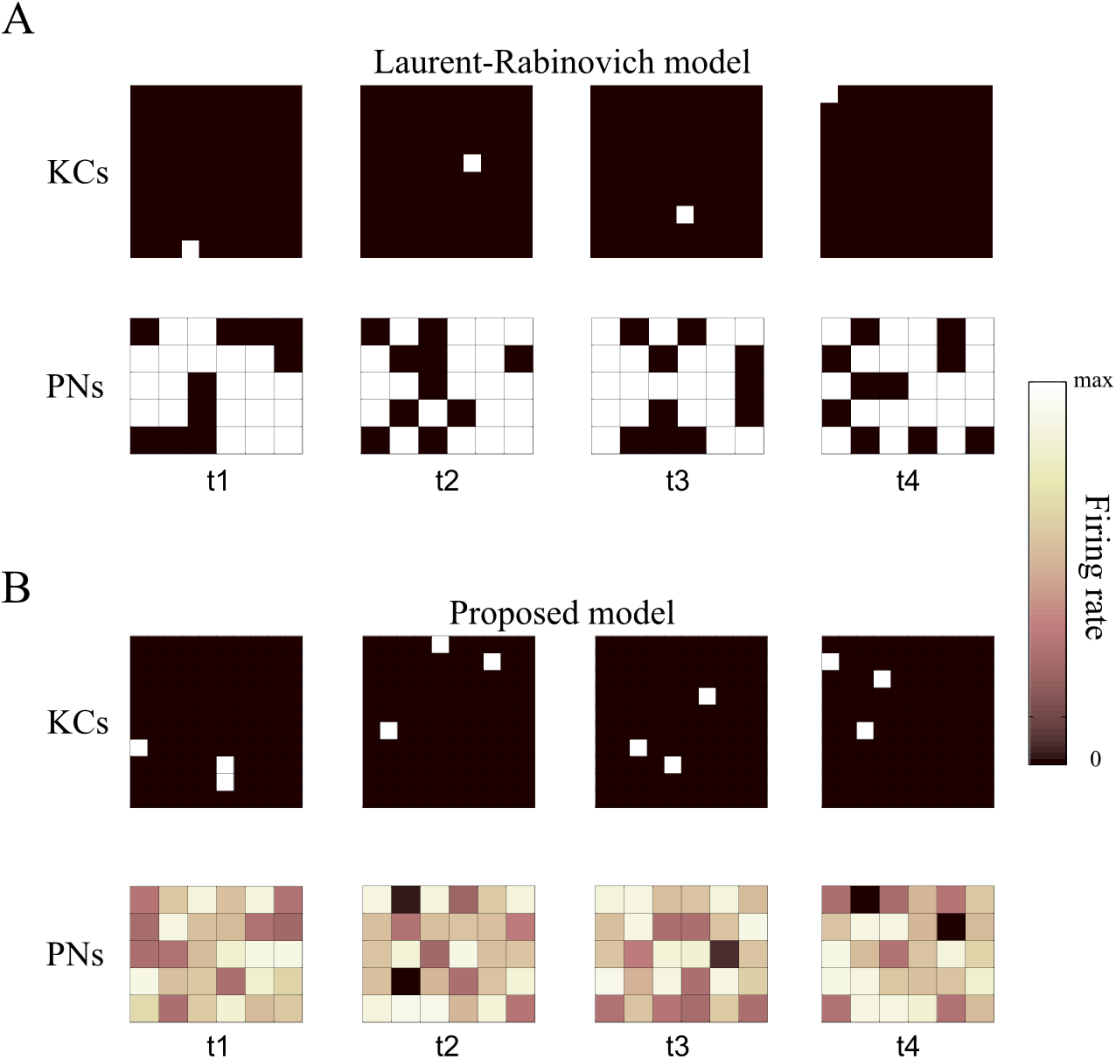
PN output of our model compared to the Laurent-Rabinovich model. Firing rates of 100 KCs and 30 PNs in mushroom body and the antennal lobe, respectively, for the proposed model and an implementation of the Laurent-Rabinovich model. Each panel represents the activity of the KC and PN populations at times t1, t2, t3 and t4, in which the clusters of the sequence are at their maximum value (see Fig 5), and each square in a panel represents a neuron (10x10 for KCs, 6x5 for PNs). (A) An SHS model, as presented in [11], used for the KC population, plus a random 1 KC to 20 PNs projection. (B) Data generated by the proposed model, with clusters of three KCs each.

To illustrate the difference between our approach and the previous work in [11], in Fig 2A we show data of KC population activity as would be expected from the [11] model (Eq 1). We use a projection rate of 1:20 into the PN population (i.e. one KC connects to 20 PNs, equivalent to connecting each PN to around 60 KCs), to create the closest resemblance to the experimentally observed patterns in the PNs. This should be compared against Fig 2B, where we show KC population activity generated by our model. It can be seen that the resulting PN activity patterns are denser than in the Laurent-Rabinovich model and qualitatively more similar to experimentally observed PN activity. See also below. In addition, in our model, KC activity is sparse but not restricted to exactly one neuron active at a time. By using clusters the model can produce more varied PN activity, closer to that reported, for example, in [24] for mitral cells and the more nuanced firing rates curves of PNs shown in [6,7,25].

#### A combined model of principal neurons and Kenyon cells

**Fig 3:**
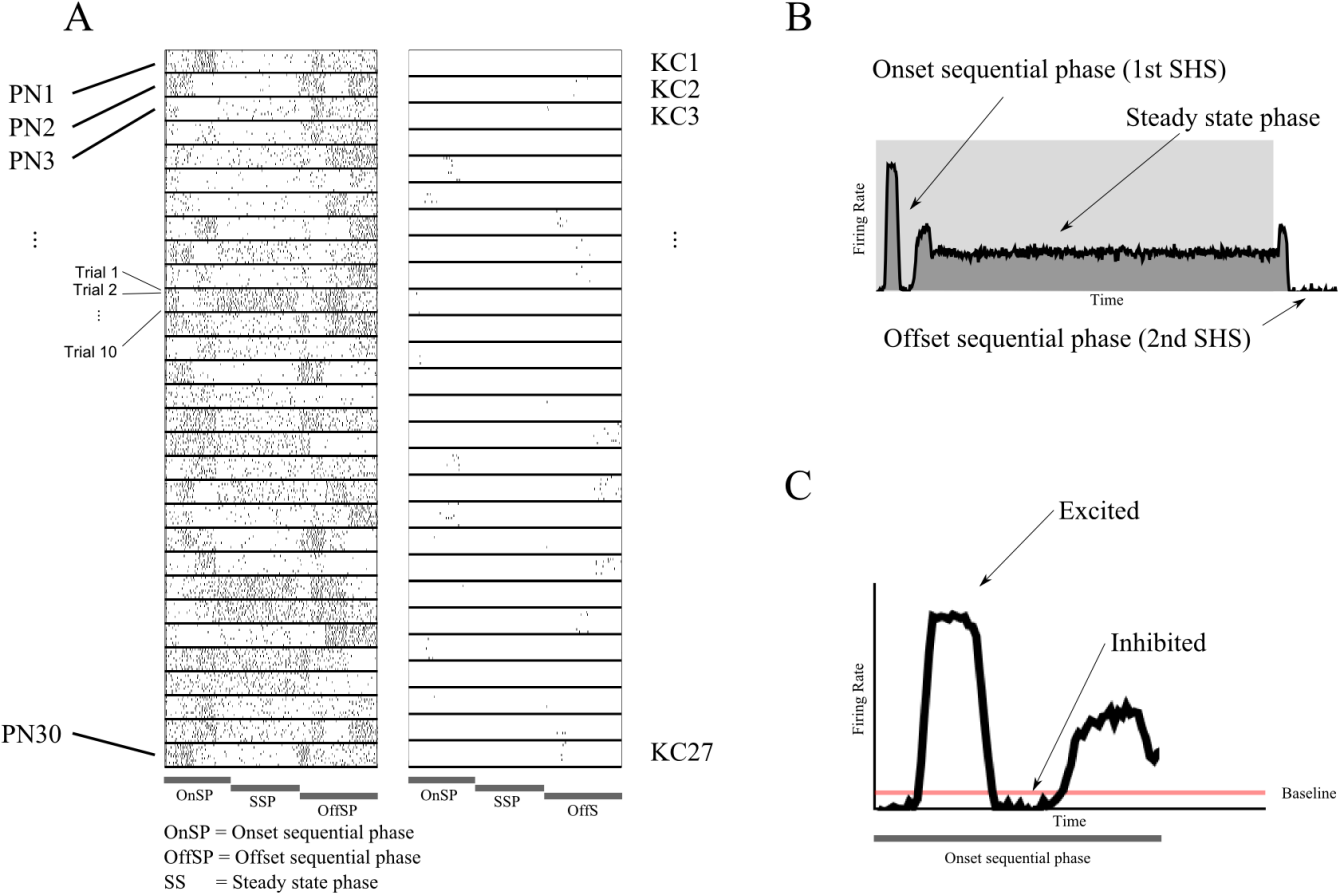
Illustration of experimental findings. (A) Raster plots for the full response (three phases) to a stimulus of PNs (left) and KCs (right) using a modified version of the generative model (see Methods for details; also see the S1 Text). This modified version was only used for the generation of this figure and no inversion was performed on it. Although the model consisted of 30 PNs and 100 KCs, most KCs were inactive during the simulations. Only the 27 active KCs are shown. The simulation was run for ten trials and the responses of different neurons are separated by thick black lines. For the PNs, periods of inhibition and excitation can be observed. During the steady state phase the population no longer evolved. KC responses are very sparse, with only a few spikes per KC throughout a trial. (B) Instantaneous firing rate for a sample PN. (C) The periods of excitation and inhibition (with respect to a baseline) during the dynamic phase for the PN in B. The baseline shown is calculated as half the minimum PN activity that the generative model can produce before it is zero.

To combine the PN and KC levels in the model, we used two experimental findings (reviewed above, also see Fig 3 for an illustration): (1) A single PN can respond to a stimulus in a multiphasic way during the dynamic phase (see Fig 3C for an illustration). (2) The PN population responds to a stimulus with a sequence of dense spatial patterns, whose order is reproducible across trials, even if the exact timing varies (see Fig 3A). To accomplish this, we used a linear, one-to-many projection from individual KCs to the PNs to create the activity of the PN population during data generation (a connection direction that is later inverted by Bayesian inference; see below for details). Experimental evidence for feedback from the KCs to the PNs has been found, for example, in *drosophila* [26]. The PNs are connected randomly with the KCs under the constraint that each KC connects with a fixed number of PNs via an observation equation. This equation is:

Y=ΘX+Γ 2

where *Y* is the *N*×1 PN firing rates vector, Θ is the *N*×*M* observation matrix, *X* is the *M*×1 vector from the Lotka-Volterra system, representing KC activity, and Γ is Gaussian noise. The observation matrix Θ connects each KC with 20 random PNs. For details, see Methods.

Eqs 1 and 2 as described in this and the previous sections are used to form what is called a generative model (see Methods for details), which is the basis of the dynamics of the KCs and PNs in our model. The connections contained in the generative model are used as a starting point by the Bayesian inference (see below) to build the full set of interactions between the neurons in our model.

Additionally, we used this generative model to generate the synthetic data that were used as input to the model in the following sections.

#### Bayesian inference

As third extension, we combine a generative model (built with Eqs 1 and 2; see Methods) with a Bayesian online inference scheme. This enables the model to read PN activity and infer the state of the KCs that is consistent with the PN activity, effectively making the PNs output to the KCs, as physiologically expected, inverting the projection from the KCs to the PNs of the generative model. This approach is useful for three reasons: Firstly, this extension makes the model an online recognition model. By online we mean that the Bayesian inference used here reads at each time step the current PN input and changes the KC states. This emulates precisely the online decoding of an insect brain. In particular, as we will show, it enables the model to recognize stimuli online and rapidly, i.e. before a full odor stimulus has been played to the model. Secondly, it provides the model with robustness to noise from several sources, e.g. neuronal noise at both the PN and KC levels. This robustness is important because it explains how a model based on potentially noisy neurons can recognize a stimulus accurately and reliably. Thirdly, it adds cluster-specific KC connections necessary for robustness and intelligent coincidence detection (see below) and connections from the PNs to the KCs. These PN-KC connections fit experimental observations [27].

Importantly, the Bayesian inference approach allows us to use a single, fixed connectivity matrix which encodes all previously stored sequences. The dynamic input to the system, i.e. the PN population activity, is used by the Bayesian inference to determine which of the stored sequences is the most consistent with the currently observed PN activity. This adds neurobiological plausibility as one would not expect (as for example in Eq 1, [11]) that the process of odor recognition would change the connectivity between neurons on a short time-scale. The online recognition is also useful for modelling ecologically valid scenarios where the actual input deviates from the expected (stored) sequence. As we will show, the model can, depending on the size of a deviation from the expected input, either explain the deviations away as sensory noise, or rapidly switch to another internally stored odor sequence, which may explain the current input better.

Through Bayesian inference, the KCs’ role in the proposed model is consistent with the traditional view of insect olfaction, in which the KC population reacts to the activity in the antennal lobe with a pattern of on/off activity, unequivocally identifying the odor perceived [28]. In addition to the on/off patterning discussed in [28], we were able to model the temporal dynamics of the response to an odor in both the antennal lobe and the mushroom body. This temporal patterning can be considered an extension to the ideas presented in [28]. Furthermore, evidence of these patterns has been found for the PNs [4,7] and preliminary evidence for the KCs [5,6].

**Fig 4:**
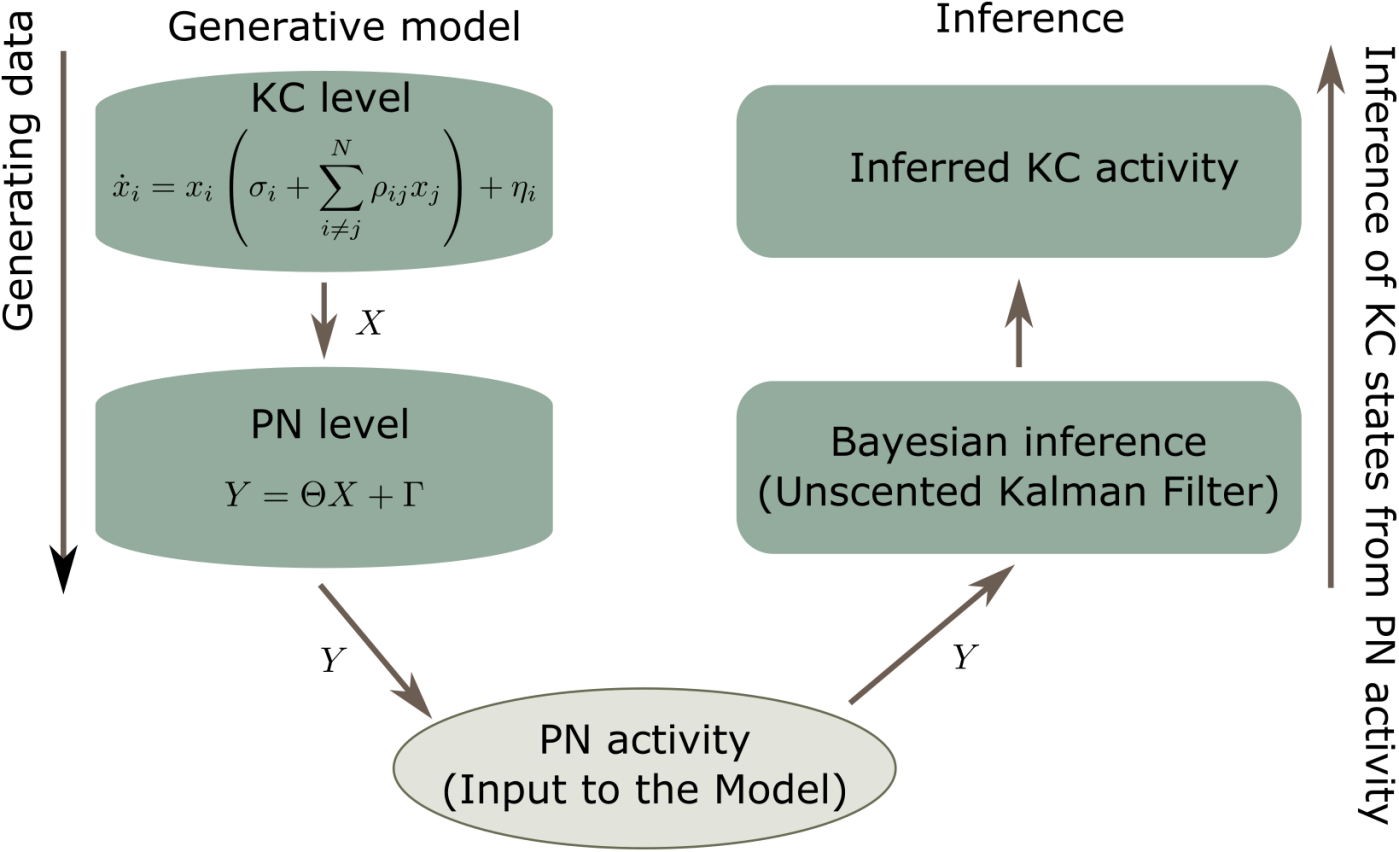
Diagram of the proposed model. The generative model was used in two ways: first, to generate the PN data that was used as input to the Bayesian inference; Second, to describe the internally expected dynamics of the PN and KC populations. Top left: Specifically, the generative model uses the Lotka-Volterra equations to generate activity of the KC population. Bottom left: This activity was projected into the PN population via Eq 2 giving synthetic PN activity that was used as input to the Bayesian inference. Bottom right: Through the unscented Kalman filter, the PN activity observed was balanced with the expected dynamics of the KCs (prescribed by the generative model). Top right: This enabled the model to infer KC activity that is consistent with the PN input data.

In summary, the resulting model aims at decoding PN activity using dynamic expectations about KC activity; see Fig 4.

#### Model parameters used for simulations

In the simulations below, we used the same parameters both for generating the synthetic data used as input to our model and for the generative model for Bayesian inference. We used populations of 100 KCs and 30 PNs with a KC:PN connectivity ratio of 1:20. The sizes of the populations were chosen in order to have a much larger number of KCs than PNs while keeping the population sizes small for computational efficiency. We used four clusters per sequence and three KCs per cluster. As a proof of concept, we typically embedded two odors, for computational expediency, into these KC and PN populations. This means that per simulation at most 24 KCs were used for representing the two odors, although the exact number changes from simulation to simulation because the KCs which represent an odor are selected randomly from the population and a KC may be involved, as found experimentally, in representing both odors.

In the generative model, there were four types of connections (see Fig 1), all of which are one-directional. For a cluster size of *N* KCs, these are: (i) Low-inhibition connections from the neurons in a cluster to those of the next cluster in a sequence, with weight 1/(10*N*). (ii) Medium-inhibition intra-cluster connections, among the neurons within a cluster, with a weight of 1/(3*N*). (iii) High-inhibition connections from neurons in a cluster to those in the previous cluster in a sequence, with strength 2 / *N*. (iv) Highest-inhibition connections between neurons not in any of the three past categories (e.g. a neuron and those that do not share any odor representation with it), with weight 2. Notice that the fourth type of connection is not normalized by the size of the clusters. The connections from the KCs to the PNs are selected randomly and the weights are all set to one.

Unless otherwise stated, data generated with the generative model were obtained with additive Gaussian noise in the PNs (Eq 2) with a signal-to-noise ratio (SNR) of 10, defined here as the activity of one PN when only one of its KCs is active, divided by the covariance of the noise. We used no noise in the KC dynamics (Eq 1) for data generation.

**Fig 5:**
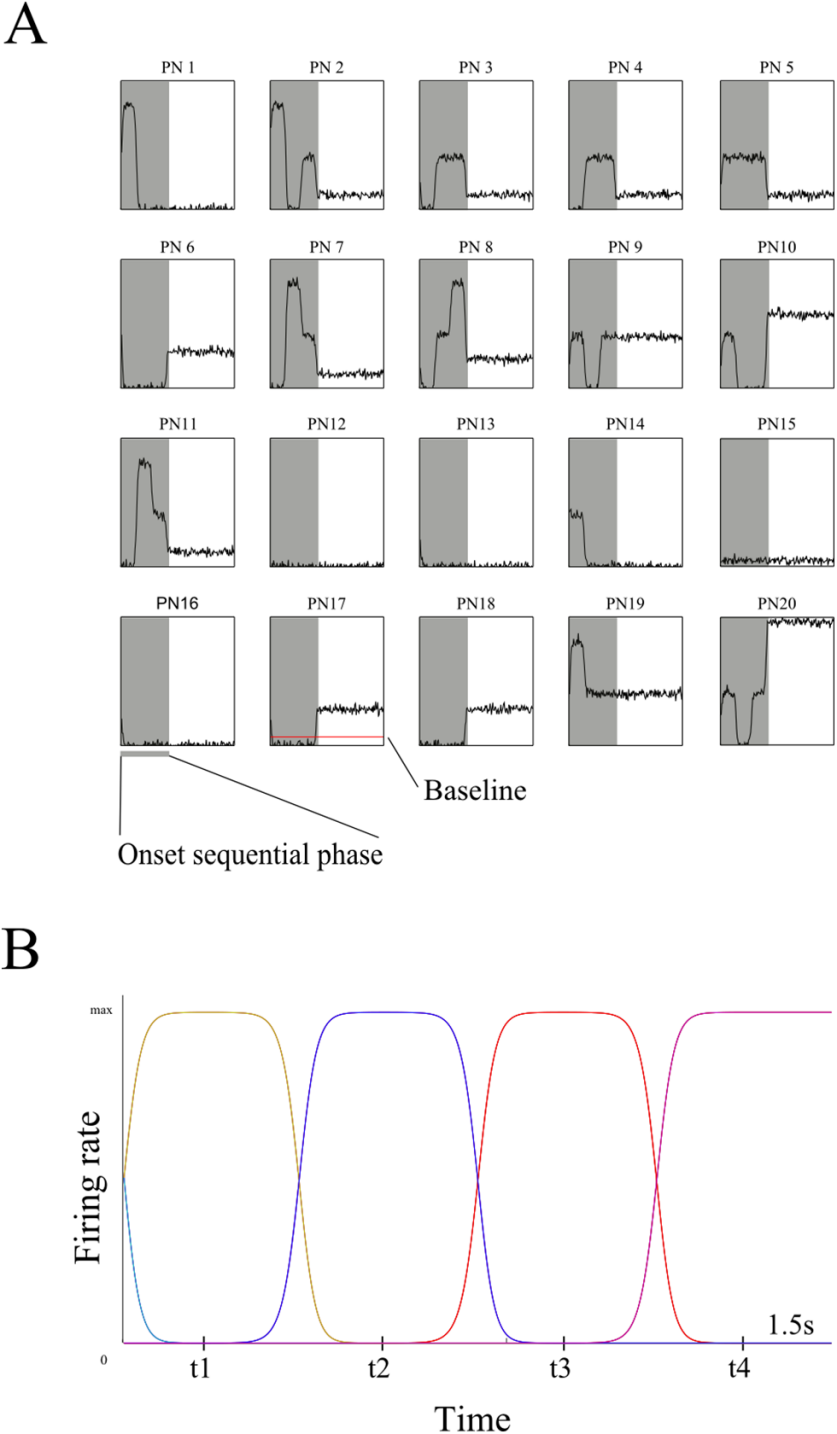
Example activity of the generative model’s PN-KC network. Y axis represents instantaneous firing rate, X axis is time. (A) Activity of 20 sample PNs that all connect to the same KC, showing epochs of excitation and inhibition. The shaded area is the onset sequential phase; the rest is the steady state phase. (B): Activity of the KCs during the onset sequential phase where each colored line represents a cluster of three KCs. In total, 15 KCs are shown (five clusters), with maximum firing rates around times t1, t2, t3 and t4. As in Fig 3, the baseline shown is calculated as half the activity level of a PN when only one KC of those connected to it is active.

In Fig 5A we present synthetic data generated by the generative model during the onset sequential phase for twenty representative PNs which connect to the same KC. It can be seen that the activity of individual PNs can be classified according to the succession of inhibition and excitation epochs through time, as found in experimental data [27]. The activity of the KC population is shown in Fig 5B. This sparse activity is representative of what has been found experimentally: only a few KCs are active at a time in odor-dependent clusters that activate together, while the rest of the population (i.e., those KCs not responsive to this particular stimulus) remains completely inactive.

Note that before choosing these model parameters, we investigated cluster sizes from two to as many as ten KCs per cluster, and sequence lengths of up to 20 clusters per sequences. We found that these variations had no impact on the qualitative results shown below, i.e. the proposed model is robust over variations in cluster size and sequence length. In addition, the model also seems to scale with the number of KCs in total: We successfully simulated models from 50 to 1000 KCs with a proportional number of PNs. We do not report these results because they were qualitatively similar to the results presented here.

Since the PN population has been found to change its activity pattern completely every 50–300ms [7,29], we selected four clusters per sequence, which amounts to around 300ms per cluster in response to an odor. Lacking experimental results for the number of KCs that activate together, we settled on three neurons per cluster.

### Simulations and replication of experimental findings

In this section, we will show the usefulness of the model in explaining how odor recognition based on the PN-KC hierarchy can be both rapid and robust. In addition, we will present a mechanistic explanation for several key experimental phenomena. There are five sections, where each addresses a specific experimental aspect. Firstly, we show how the model performs fast odor recognition, in accordance with experiments. Secondly, we show that the experimentally established steady state phase of the odor response can be explained by our model as a prolonged activation of a single neuronal sequence element. Thirdly, we demonstrate the robustness of the model against several types of neurobiologically expected noise. Fourthly, we show how the KCs in our model behave as intelligent coincidence detectors of both PN and KC activity. Fifthly, we show that the model KC trajectories reduced to three dimensions look precisely like their experimentally observed counterparts. This finding in particular indicates that our model captures a fundamental aspect of KC activity measured during insect odor recognition.

#### Early odor recognition

In experiments, it has been found that the PN trajectories in the phase space of PN firing rates are maximally separated from each other during the onset sequential phase, facilitating odor discrimination [7]. In both insects and rodents the reaction time for odor recognition is much shorter (< 200ms) than this 1.5s-long first phase [6,30–32]. What is the function of the full 1.5s dynamic response to an odor? The answer might be a speed-accuracy tradeoff [30], which has been observed experimentally in rodents [30,33]; in this case, the 1.5s neuronal response may allow the brain to perform integration of information over time, improving accuracy in hard discrimination tasks, where the two odorants to be discriminated are presented in solutions with varying concentration ratios between them. In these tasks, the representations in the PNs of the two odors are very similar. Here we show that in our model, consistent with experimental results, the reaction time increases with harder tasks while maintaining a given accuracy level. We also find with our model that the reaction time is very short for most tasks.

To show this, we embedded two odor representations (sequences) in a system, with the system’s parameters set as described in section ‘Model parameters used for simulations’ and used this to generate PN data to provide input to the model. We performed simulations with increasing degrees of task difficulty. To control the task difficulty, we changed the similarity between the two embedded odor representations, as is done experimentally [31]. To assess model performance in the recognition task, we compared the reaction times (see Methods). Choice comparisons were uninformative because the model performed mostly at ceiling for all tasks.

The easiest task for the system was when the two sequences were similar only at the KC level. We implemented this by defining the first clusters for the two odor sequences such that they shared two out of three KCs. When playing one of the two odors to the model, we found that the model identified it correctly and rapidly (<60ms). This was expected because a sequence difference in a single KC expresses itself as a massive difference in up to 20 (out of 30) PNs in the projection pattern of the two sequences.

**Fig 6:**
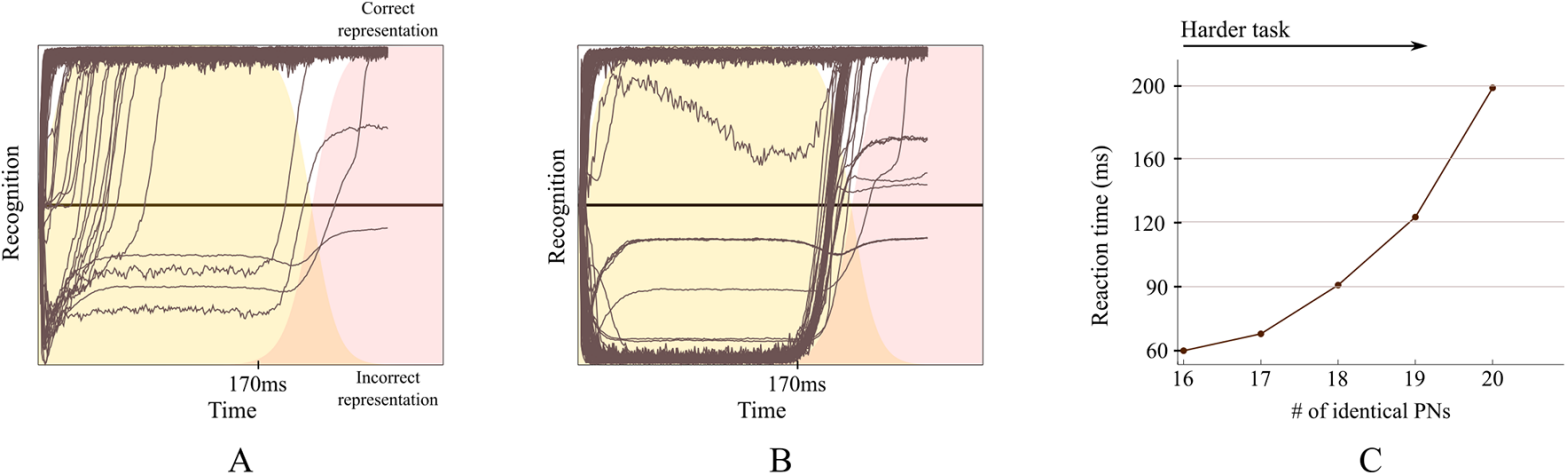
Performance of the model in difficult tasks. The connectivity matrix has two sequences which were similar to each other. (A-B) Each single line represents one trial (out of 100 trials) simulated from the model. The Y axis represents the recognition variable, which is calculated from the difference between the Euclidean distances from the observed KC activity to the correct one and from the observed KC activity to the incorrect one (see Eq 6 in Methods). If the KCs are displaying the correct representation (encoding the displayed odor), the corresponding line is near the top. If the KCs are displaying the incorrect representation (i.e. the other sequence stored in the system), the corresponding line is near the bottom. Lines near the middle are as close to the correct representation as to the incorrect one. The activity of the clusters in the expected (correct) sequence is in the plot background as a time reference to show that many single trials quickly jump to the correct representation after the second cluster in the sequence starts: The shaded areas (2 colors) represent two clusters in the sequence (as in Fig 5B). (A) Results for a task in which the PN data for the two stored sequences are very similar during the first cluster: Only three out of twenty PNs are different for the two sequences during the first cluster. Due to noise, the KC activity represents sometimes the incorrect odor for a brief period of time. (B) Results for a task in which the PN data for the two stored sequences are identical during the first cluster but dissimilar for the rest of the odor. During exposure to the first segment of the odor, the representations on the KCs are correct around 50% of the time, as expected, consistent with random chance. When the second cluster in the sequence begins (at ca. 170ms), the KC activity quickly jumps to the correct representation. (C) Average reaction time of 100 trials plotted against difficulty of the task (as defined with the number of PNs that belong to the representations of both odors; see Methods). The PN representations of the two stored sequences are similar during the first 170ms; afterwards, they diverge and become easily identifiable. The more similar the PN representations of two stored odors are during this initial period, the longer it takes the KCs to identify it. The maximum reaction time, around 200ms, corresponding to the case in B, is obtained when all 20 PNs are the same. The case in A corresponds to 17 identical PNs.

To make the recognition task more challenging we used similar KC clusters with two shared KCs between odors. However, differently from the easy task, for the differing KC we employed PN representations that were more similar than in the easy task. The similarity is expressed as the number of PNs that are connected to both of the differing KCs in the two clusters, where each KC connects with 20 PNs. For example, an easy task of this type is when there are only three PNs out of 20 (see Fig 6A) that connect to both KCs and the hardest when there are 20 (see Fig 6B). When 20 (out of 20) PNs are shared between the two KCs, the task is actually impossible to solve because the two odors are represented by exactly the same PNs during the first cluster. With increasing difficulty, we found that the system required, on average, more time to identify the correct sequence (i.e. the correct odor); see Fig 6C. For the easiest task, the mean reaction time was 60ms, which increased for harder tasks, where more PNs were shared between the two different KCs. For the hardest task, the model could not solve the task during the first cluster (50% accuracy) but had to wait for the onset of the second cluster (ca. 170ms) to then solve the task with a mean reaction time of 200ms.

Note that these simulations present a worst-case scenario to the model because the difference between the two odors was right at the beginning of the odor sequence. If these differences are found rather towards the end, the high similarity would not impede or slow down recognition at all: We found that, if the two embedded representations are different enough at the beginning (i.e. their initial cluster is different), being identical later on has no effect on the performance of the system, as the correct representation has already been found and the system sticks with this sequence. This may be a general strategy for odor recognition: represent odors such that they are most dissimilar at the beginning of the response. As we have found in our simulations, minute differences among odor representations, as expressed in PN activation patterns, are sufficient to recognize the correct odor rapidly, with a high probability. Such an encoding strategy may explain experimental results reporting the lack of evidence supporting a speed-accuracy tradeoff [31,34]: two odors that, on average, have highly similar representations in the brain might be different enough at the beginning to allow for rapid discrimination.

#### Steady state phase

The second phase in the PN activity in response to a stimulus is a steady state phase, in which the temporal evolution has been found to halt until odor offset [7]. During this phase, the PN activity shows sustained firing at a lower average firing rate than during the first dynamical phase [7]. See Fig 3B for an illustrative example. The KC activity follows this trend, having lower sustained activity during this phase [5]. This phase is typically observed when the insect has been presented with a stimulus for longer than ca. 1–2 seconds and the PN response has been found to contain information about the identity of an odor. However, during this phase, it is harder to distinguish between the representations of different odors than during the first dynamical phase of the response [7].

The function of the steady state phase is currently unknown, but based on the proposed model we suggest an explanation that is both mechanistic and functional. As the model is based on neuronal sequences (Eq 1, also see Methods), we explicitly avoided model components like fixed point attractors which may serve as a relatively direct explanation of a steady state phase. This means that the proposed model should have difficulties to represent a steady state phase. However, when we created PN data with the typical sequence of onset sequential phase followed by the steady state phase, we found that this steady state phase can be represented easily be the model: The steady state phase is simply the consequence of the odor-encoding KC cluster sequence having run out of sequence elements to represent this specific odor over time.

**Fig 7:**
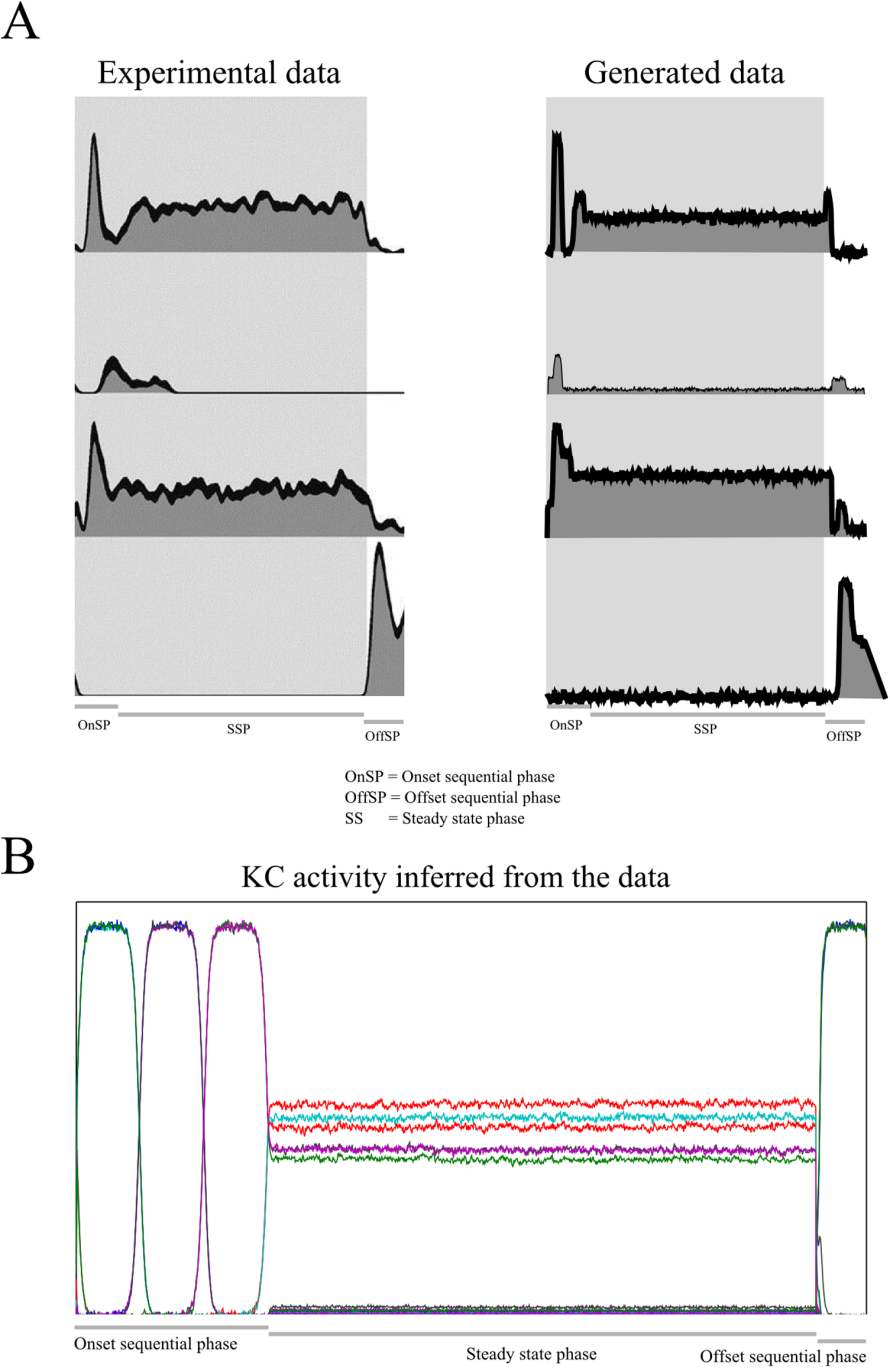
PN and KC activity for the full response to a stimulus. (A) Sample of four PNs’ activity throughout the full response. Left: experimental data as reported in [7]; Right: data generated by our model. The qualitative behavior of the PNs observed in experiments can be matched by our model by choosing an appropriate observation matrix. (B) Bayesian inversion of the model: Inferred KC activity as a response to the PN data generated from the full response shown in A. Each color represents a cluster of three different KCs. It can be seen that the inferred KC activity displays the three phases of the odor response, although the generative model given by Eq 1 includes only the sequential dynamics.

To illustrate how this steady state phase emerges in our model, we generated PN data corresponding to the full stimulus response with the three phases (Fig 7A, see Methods for details) and used it as input to the Bayesian inference. It can be seen that this PN input is remarkably similar to that observed experimentally.

The model KC activity, in response to this PN input, is clearly divided into the three phases, as found experimentally when recording data from PNs (Fig 7B). In particular, the KC responses of the model show the steady state phase of the response with a lower firing rate than the sequential phases. Remarkably, the transitions between phases (from sequential to steady state and back to sequential) are reproduced by the model, even though we did not explicitly encode such a mechanism in the dynamics of the KCs in our model that would account for this behavior.

The explanation for this finding is that during the steady state phase, the last element in the sequence lingers on as long as the stimulus is presented. Our model explains this phase as just another element of the sequence that lasts longer than the preceding elements. Thus, it contains as much information about the odor identity as any other spatial pattern of the response. However, the information encoded in the temporal evolution is missing, which would explain the experimental finding that the steady state phase contains information about the odor stimulus but not as much as during the onset sequential phase. This model finding suggests a highly efficient representation of odors in insects because any odor duration can be represented by stretching the last element of the odor encoding sequence in time. Our model suggests that the insect odor system may be primarily geared towards fast recognition of any occurring odor and probably uses the last element of the sequence as a device to represent the constant odor input. This model behavior is due to the Bayesian inference technique: The temporal stretching of the last cluster of the sequence is the model’s successful attempt to fit its internal expectations (sequential dynamics) and the external input (constant). These results suggest that KC function in the insect antennal lobe may be largely aimed at representing neuronal sequences. The proposed model suggests a simple mechanism for how the qualitatively different three phases [7] can be encoded parsimoniously.

#### Robustness against noise and time-jitter

As odor recognition in an insect has to be robust against adverse conditions in nature, a model should emulate this robustness (i.e. perform well even under adverse conditions). We tested this by making odor recognition difficult for the model by exposing it to noise and interferences. To do this, we used three different types of noise and interferences: (i) In nature, odors may be perceived with a low signal-to-noise (SNR) ratio, e.g. because the concentration of odor is low. We will test whether the proposed model can deal with such low SNRs. (ii) The representation of an odor in the PNs has been found to be in the form of a trial-variant sequence of spatial patterns, whose exact timings have been found to change for different trials of the same insect-odor combination [7]. Therefore, the insect brain’s recognition can be assumed to be robust against time jitter in the representation of an odor. We will test whether the model has the same robustness for time jitter. (iii) In experiments, insects were presented with an odorant for some time; before and after this stimulus, the insect is typically presented with clean, odorless air [6]. This brings the PNs and KCs back to baseline activity, in which they are not having any discernible response. In nature, such ‘bringing back to baseline’ may not happen. The brain must be able to recognize an odor that is presented right after another; in terms of neuronal activity, it means that recognition should be robust against the activity of KCs being different from baseline at odor onset. We will test whether the model can handle these deviations from a baseline state when recognizing odors.

**Fig 8:**
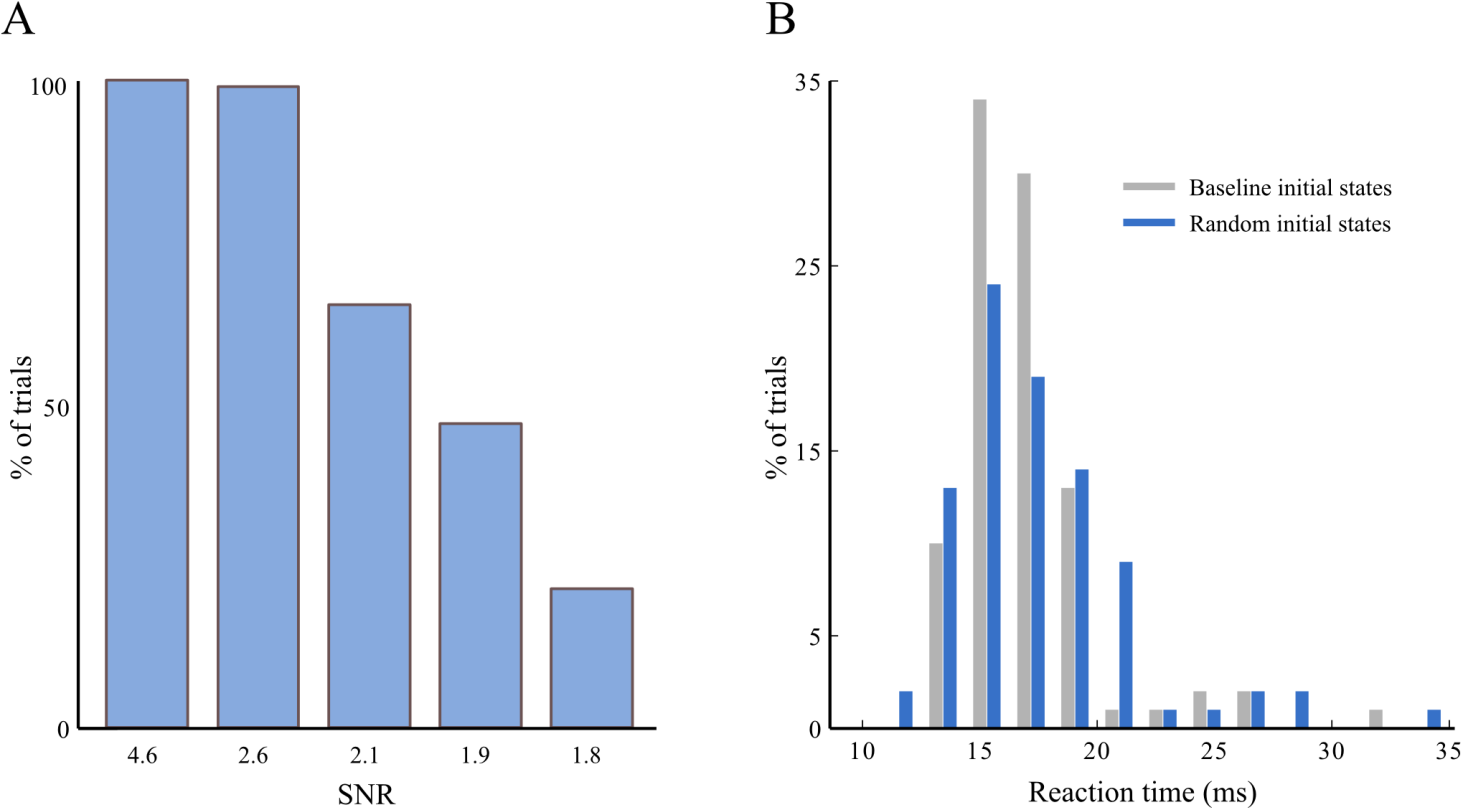
Performance of the model under adverse conditions. (A) Simulations to test robustness against noise in the PNs. Percentage of correct trials (i.e. trials in which the model displayed the correct KC representation) for low SNRs. For these simulations, we added white noise of different variances to all PN activity. The model is said to display the correct response if the Euclidean distance in the firing-rate-space between the inferred and expected (i.e. used to create the data) KC responses is smaller than a threshold (see main text). A decrease in performance can be observed only for SNRs smaller than 2.6. (B) Simulations to test robustness against KC states not at baseline at the onset of an odor. Histogram of reaction times (see Methods) for trials with initial KC states set to a baseline (light gray) or a random state (blue). These reaction times were calculated for two completely dissimilar sequences with high SNR. Therefore recognition is rapid. The reaction times for trials with random initial conditions are slightly more varied and the mean reaction time is 19ms, as compared to 17ms when the initial KC activity is at baseline.

To test the model against neuronal noise, we ran all our simulations with white noise in the PN data. We used several levels of signal to noise ratio (SNR): 4.6, 2.6, 2.1, 1.9 and 1.8, which cover the spectrum between low noise, with ceiling recognition performance, and high noise causing highly degraded performance. For each of these noise levels, we computed the number of correct trials (out of 100) by using a threshold on the Euclidean distance between expected and inferred KC activity, as was done for the reaction time (see Methods). This conservative way of computing a correct response may be interpreted as counting a response as correct only if there is certainty on the response. This explains why we found for low SNRs performance levels of below 50% (which would be chance level in a forced-response paradigm). We found that the combination of the Lotka-Volterra equations with Bayesian inference is robust against this kind of noise for low SNRs higher than 2.6 and exhibits a graded deterioration of performance for smaller SNRs; see Fig 8A.

To test the robustness against a wide range of non-baseline KC activity at odor onset, we randomized the initial states of the KCs in our model with a uniform probability distribution between zero (inactive) and one (the maximum normalized firing rate). We found that this random initial activity slightly impedes the accuracy of the response at the KC level when measured with the conservative threshold approach above (<6% of cases, against <0.1% for baseline initial states). The mean reaction time for the non-baseline KC activity is slightly delayed and the variance of reaction times is slightly increased (see Fig 8B). In principle, this observed robustness against deviations of KC activity from baseline allows the model to process odors even when they follow each other quickly such that the odor recognition has no time to relax to baseline between odors. The finding that there is a slight increase of ‘failed’ recognition trials (from <0.1% to 6%) when the system is not in its ‘ready’ baseline state, is interesting in light of similar findings in cognitive neuroscience where perceptual performance has been found to depend on the internal state of participants [35,36].

**Fig 9:**
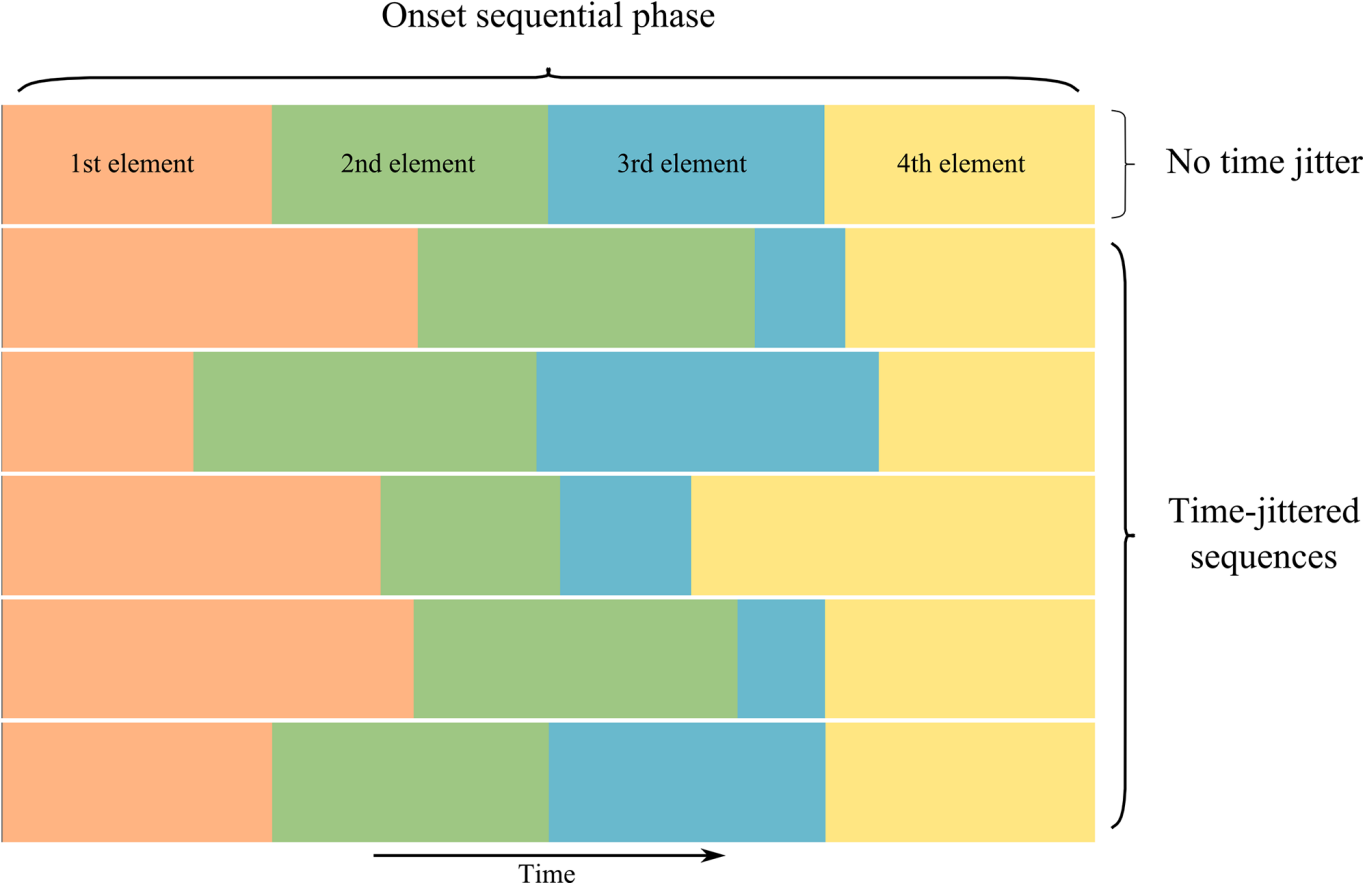
Time-jittered KC activity from the model. Using one connectivity matrix, we generated PN data with no jitter and used that data to create time-jittered PN activity. The KC activity output by our model is shown: each row represents a sequence displayed by our model; each color represents a cluster in the sequence. The first sequence (top) has no time-jitter in the input. The following sequences are time-jittered, i.e. the time each cluster is active is different. It can be readily seen that the Bayesian inference has no trouble handling time-jitter that cannot be generated directly by the generative model.

To test the robustness against time-jitter in the odor presentation by PNs, we created time-jittered PN data by processing the responses generated by the Lotka-Volterra equations to change the time each cluster is active during the sequence. In Fig 9 we show time-jittered example sequences in the KCs obtained with our model. We found no discernible effect of the time-jitter on the obtained responses from our model, i.e. mean reaction times are statistically not different and performance is at ceiling. That is, the model KCs display the expected (time-jittered) response to the stimulus, closely following the time jitter of the data. This is quite remarkable because the generative model prescribes a specific duration of each cluster response. The reason why the inferred KC activity can closely follow the time-jittered input is that the Bayesian inference can easily delay or speed up KC activity to match the time-jittered PN activity. This finding indicates that the insect brain may use similar computations as used by the model to adjust KC activations to the ongoing time-jittered PN activity. Importantly, such a computational strategy to deal with time-jitter means that the internal model of the insect as encoded by the KC dynamics can be kept simple as typical deviations imposed by nature can be dealt with due to the robustness of neuronal sequences against such deviations.

#### Intelligent coincidence detector

It has been found that a KC activates when the number of active and connected PNs surpasses a threshold [5,8,37]. For example, in honeybees this threshold was calculated to be around six out of the ten PNs that connect to a single KC [8]. This sort of coincidence-detector mechanism is thought to provide the brain with robustness against noise by responding to a range of PN responses instead of just a specific one [38].

Here, we will show that our model implements a coincidence detector which goes beyond simple thresholding of active PNs. We suggest that a similar mechanism may be used by the insect brain. The idea of simple thresholding is that the KCs evaluate how many of its connected PNs are active and only gets activated if a specific threshold of active PNs is surpassed. The way the proposed model improves on this intuitive procedure is as follows: Since each KC is part of a cluster, the activation state of the other within-cluster KCs and the temporally preceding KCs is highly relevant for the activation state of that KC. For example, if in our model a KC sees only a limited number of its PNs active, but its within-cluster KCs are fully activated at the same time and transmit this information by lateral connections, this is a strong sign for the KC that the low number of active PNs is just due to noise and it should activate as well, despite the contradicting activity of its input. However, if the within-cluster KCs are not activated, and a high number of PNs is inactive as well, this would be a clear sign that a KC should not activate. In other words, for the proposed ‘intelligent’ coincidence detector, a KC receives both bottom-up sensory and lateral information. This intelligent coincidence detector is automatically done during recognition. A simple threshold detector would only receive bottom-up information.

**Fig 10:**
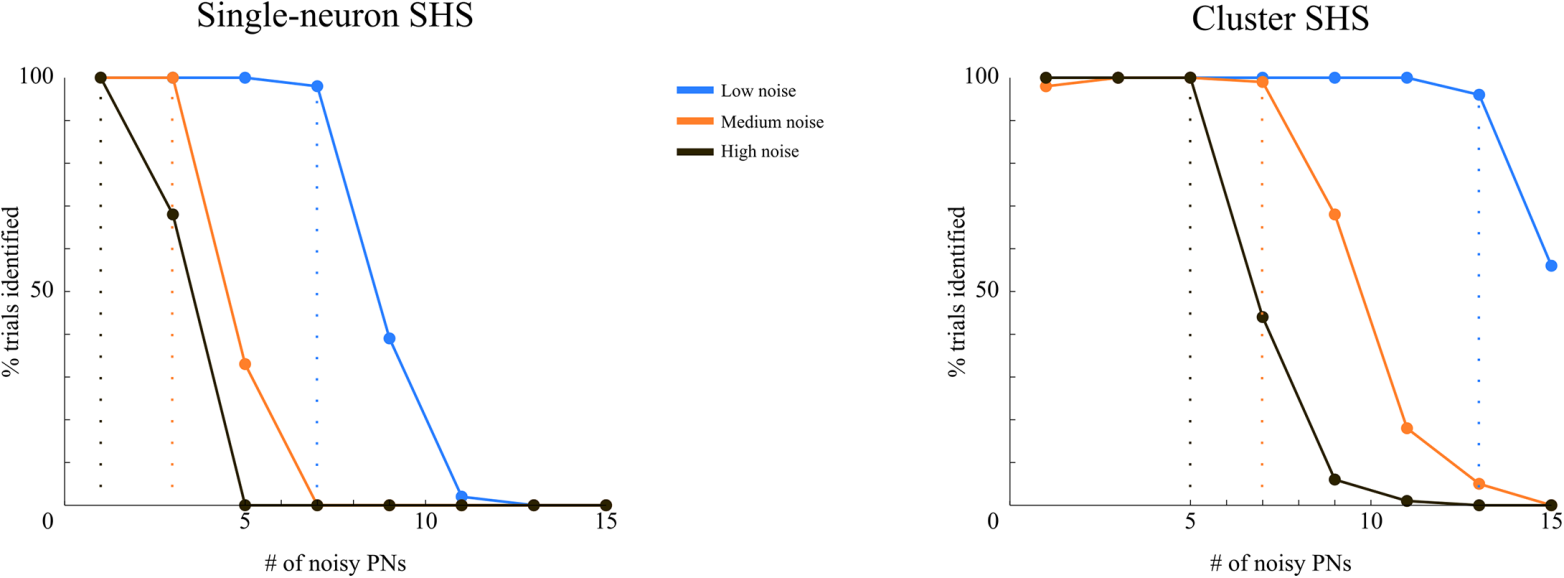
Performance of the model with noisy PN input. The performance of our model was measured in terms of displaying the expected sequence in response to the input while a number of PNs that connect to a single KC display large amounts of noise. Three noise levels were used (different colors); with high noise (SNR of only 1), the activity of a PN is mostly noise-driven, because the fluctuations due to noise are comparable to activity of the noise-free PNs. With an SNR of 2 (called here low noise), the noise variations are about one third of the maximum noise-free PN activity. Medium noise had an SNR of 1.5. On the x-axis, we plotted the number of noisy PNs; on the y-axis, the percentage of trials in which the KCs displayed the correct representation (100 trials for each number of noisy PN/noise combination). Left: performance of an inverted Single-neuron SHS generative model. We used a modified version of our generative model, in which single KCs, as opposed to clusters of KCs, are activated sequentially. This is then projected to the PNs as described for our proposed model and inverted with the described unscented Kalman filter. Right: performance of proposed model with Cluster SHS. The large differences in the performance of the two models can be attributed to the within-cluster lateral input that the KCs receive (i.e. intelligent coincidence detector) in the Cluster SHS model: The performance of the single-neuron SHS model degrades for smaller number of noisy PNs, for all noise levels. In particular, for high noise rather small numbers of noisy PNs lead to poor performance.

The improvement in recognition performance of an intelligent coincidence detector as compared to a simple one can be seen in Fig 10. In Fig 10A we show the performance of a simple coincidence detector. To compute these results using our model we removed the cluster aspect by replacing clusters with single KCs. In essence, this single-neuron stable heteroclinic sequence (SHS) model is the Laurent-Rabinovich model combined with Bayesian inference and an observation equation for PNs. One can clearly see that recognition performance was dependent on both the number of noisy PNs and the SNR of the input. For high SNR, performance rate stayed high (i.e. > 90%) for as many as 7 out of 20 PN failures. For medium and low SNR, this threshold was 3 and just 1 PN failures, respectively. When we used the proposed cluster-SHS model (Fig 10B), the performance for high/medium/low SNR remained high (>90%) for up to 13/7/5 out of 20 PN failures. Another way of looking at this is to observe that the cluster-SHS model could still recognize at >90% performance when the single-SHS model was completely lost at 0% performance. This means that a key advantage of the cluster-SHS encoding over using just sequences of single KCs is that roughly double the amount of PNs may fail until recognition ventures below 90%.

In sum, the model acts as an intelligent coincidence detector based on lateral influence at the KC level, as implemented by Bayesian inference. This lateral influence emerges because the generative model, following experimental observations, packs multiple (here, three) KCs into one cluster. Although speculative, we here presented a quantitative functional reason why few, but more than one KC, may encode the same odor: to be better guarded against noise, either in the stimulus and/or at the PN level.

#### Projections of high-dimensional trajectories

**Fig 11:**
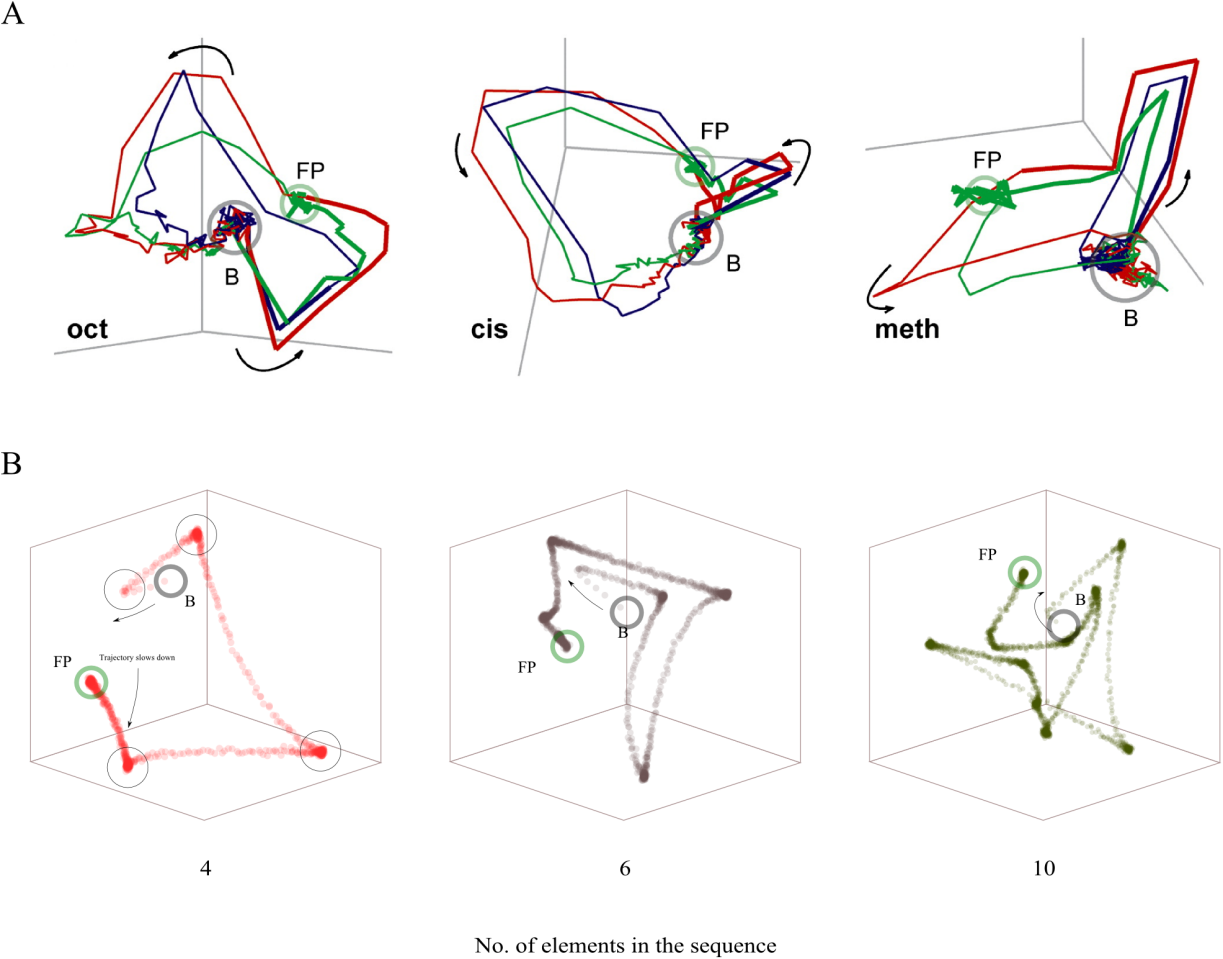
Dimension reduction of the PN response to a stimulus. (A) Data as presented in [7] showing the trajectories in the first three components of principal component analysis for three different odors (different plots) and three stimulus durations for each odor (blue, red and green for 0.3s, 1s and 3s, respectively). The dynamic phase of activity lies between baseline activity (represented by B) and the fixed point (FP), where a number of turning points can be seen, between 2 and 4 for different cases. (B) First three elements of principal components analysis of the data obtained with the proposed model of the 30 PNs during the first phase of the full response, for different number of clusters in a sequence (4, 6 and 10 clusters). The number of elements equals the number of sharp turning points in the trajectory (big circles in the left-most plot). Around each turning point, the data points agglomerate, reflecting the period in which a cluster remains active. Since we used data from only the onset sequential phase, the trajectories we present are not closed as in experimental data where the data acquisition is done up until the system returns to baseline, a few seconds after odor offset. The points B and FB, as before, represent the baseline (at odor onset) and the fixed point (end of onset sequential phase).

In this section, we show that the model, without having been designed to do so, replicates another important experimental finding in insect olfaction. With experimental data, a visualization of high-dimensional PN population activity is achieved by a reduction to three dimensions, with a minimum loss of information; see [15] for a review. This is often done using linear local embedding [6,25,39] or principal components analysis (PCA) [7,40]. The response to a stimulus of the measured PN population is typically observed as closed trajectories in the phase space of the firing rate of the observed neurons and visualized using the first three components of the reduced system, see Fig 11A. One striking feature of these three-dimensional trajectories is that there are multiple points in which the trajectory makes sharp changes in direction. From [7] one can estimate the number of these points at around 5 or 6 during the dynamic phase.

To our knowledge, a functional explanation for these sharp direction changes has not been presented yet. To address this question, we computed the same three-dimensional trajectory plots using PCA for the synthetic PN activity of our model during recognition (see Fig 11B). As can be seen from a comparison of Fig 11A and 11B, the resulting model plots look qualitatively similar to the experimentally measured plots. Although speculative, we posit that these sharp turning points represent the different elements of a KC cluster sequence. We found that, in our model, the number of turning points equals the number of clusters in a sequence (see Fig 11B). In other words, the three-dimensional trajectory plots gained from measured PNs may present vicarious evidence of a sequential activation of KC clusters. Note that we did not design the model to generate these trajectories. Rather, these trajectories are a consequence of the sequential neuronal activity of KCs expressed in the high-dimensional PN activity.

## Discussion

We modelled the time-evolving spatial patterns observed in the projection neurons (PN) of the antennal lobe and the Kenyon cells (KC) in the mushroom body of the insect brain by using a Bayesian model based on specific neuronal sequences. We were able to replicate several key experimental results, namely, the sparseness of the KC response patterns, the epochs of inhibition and excitation in the response of the PNs, the fast recognition of odors and the remarkable robustness of the model to different types of noise and unexpected deviations. In addition, as an emerging behavior of the model, we found that the model delivered novel, mechanistic explanations for two experimentally observed but yet unexplained phenomena: (i) the existence of a steady state phase in the response of the PN and KC populations and (ii) the experimentally-observed points of sudden direction changes in the trajectories of the first three response components. In addition, we found that the KCs in our model behave as intelligent coincidence detectors of PN activity, which, as we showed, helps odor recognition in the presence of noise. Finally, we showed that the model can actually decode PN firing rate activity with high recognition performance. Although a future application, we anticipate that the proposed model for insect olfactory recognition may serve as a first step towards building real-world machines for artificial odor recognition.

The proposed model was based on experimental findings and physiological data. The hierarchical nature of the odor processing in the proposed model was based on the traditional view of the way in which the insect brain processes odors [28]. The sequential activity in the PN and KC populations has been previously observed *in vivo* as part of the response to an odor [4,5,7]; additionally, experimental evidence has been found for the connections between the KC and PN populations used in the model [26,28]. In addition, the connections among KCs and among PNs in the proposed model are consistent with experimental findings [27,41].

The hierarchical processing of the proposed model appears to work in the same way as what is known about the insect brain: The traditional view is that the first step in processing the incoming signal happens in the antennal lobe, where the PNs process the information sent by the olfactory receptor neurons and transform it into a dense spatiotemporal coding. This information is sent to the mushroom body (among other targets), where the spatiotemporal coding is read. The KCs in the mushroom body exhibit very sparse spatiotemporal coding (for a review on the current view of the insect brain’s processing, see [28]).

Importantly, although the model was built following experimental observations at hand, there were three findings which concur closely with experimental findings but emerged as unexpected features. These emergent findings indicate that the model captures some of the essence of what is important in insect olfaction. Firstly, we found that the steady state response may be explained by a temporally stretched version of the last sequence element of an odor. If one wanted to model this ‘directly’, one would probably formulate a model which transitions after a sequential phase to a fixed point attractor and back to a sequence for the experimentally observed phase. We found that this is not necessary; rather the same dynamic sequence model can be used. One requirement for this to work is that one uses Bayesian inference to model how the sensory input guides the internal KC states.

Secondly, we found that the model replicated the typical three-dimensional state-space trajectories of PN activity. It came as a surprise to us how accurately the model response replicated the experimentally observed trajectories. The close match of these PN trajectories suggests that their cause is the sequential and sparse KC activity. Although speculative, the congruence of the computational model and experimental observation may be seen as evidence that the underlying KC activity is structured spatiotemporally as elements of a sequence. If this were not the case, one would not observe the sharp turns in our model plots. If the number of sharp turning points in experimental data is fixed at around four or five during the dynamical phase, as indicated by experimental results, our model results suggest that the evolution of the PN population’s response to a stimulus can be divided in four or five main stages that are well distinct from one another (each one representing an unstable equilibrium point in the KC firing rate space), making the readout (by downstream neurons) a relatively easy task. This interpretation is supported by experimental findings in locusts [7,29], where the PN population changes completely every 50–300ms.

Thirdly, we found that the model can decode (synthetic) PN firing rates with ceiling performance and is rather robust against noise and deviations (see Figs 8 and 10). One further finding was that the model implements at the KC level a smart coincidence detector on PN activity. Taken together, these findings are remarkable, because we achieved this high performance and robustness only by closely following the experimentally established blue-print of the olfactory insect brain. Without these ‘experimental’ instructions on how to build an olfactory recognition model, we would have not arrived at the high robustness of recognition. For example, by using sequences of KC clusters, as opposed to sequences of single KCs, we found a stark improvement in robustness (Fig 10). We believe that this finding is important because it suggests that the insect brain may actually use the hypothesized cluster sequence mechanism to optimize its recognition performance and robustness.

### Bayesian inference as a decoding mechanism

A key component of our model is the Bayesian inference, which helps the KCs decode the information contained in the PN activity, making the proposed model a recognition model. This decoding mechanism goes beyond a simple feedforward connection from PNs to KCs, in that it balances the expectations that the brain has about the internal dynamics of the KCs with the information contained in the PN activity. The connections that are created between PNs and KCs by the Bayesian inference through the Kalman gain (see Methods) are Bayes-optimal, in the sense that they minimize the so-called precision-weighted prediction error to obtain the best results possible.

There is an underlying assumption in the proposed model: that PNs and KCs perform computations similar to what Bayesian inference would prescribe given a generative model (of how PN activity arises from odor reception). As we proposed a firing rate model, there is no link yet to the single neuron level. It is unclear how the proposed model can be implemented by network of single spiking neurons, but the idea that the brain behaves as an optimal Bayesian observer in perceptual decision-making tasks has already been substantiated and possible neural circuits have been suggested [18,42–45]. In this work, we demonstrate how Bayesian inference, along with an appropriate generative model, can act as a decoding mechanism and explain the performance of the insect brain in recognizing odors, as well as the remarkable robustness, and other phenomena observed in experimental data.

### Metastable states as a coding mechanism

The exact way in which the insect brain’s PNs encode the incoming olfactory information is still unclear. Based on the comparison between our model and experimental data, in particular the tri-dimensional trajectories obtained with PCA (section ‘Projections Of High-Dimensional Trajectories’), we suggest that the antennal lobe encodes the identity of an odor as a sequence of a small number of metastable states in the configuration space of PN firing rates. This means that an odor is represented in the antennal lobe as a collection of points both in the phase space of PN firing rates and the transitions between these points (i.e. as a stable heteroclinic sequence). The exact number of these points may be odor-dependent, but given the speed at which PN activity changes, which has been reported from experimental data to be around 50–300ms, this number could be as low as four. Thus the odor representation could comprise, during the onset sequential phase, five metastable points and the transitions between them.

### Previous models of neuronal sequential activations

Sequential spiking of neurons or groups of neurons has been modelled before. A well-established model for spiking neurons are synfire chains, where groups of synchronously firing neurons can be set to fire in a specific and reproducible sequence, using feedfoward connections [46,47]. In another study, the parameters in the Hodgkin-Huxley model were modified to allow for the sequential firing of neurons in a desired sequence [48] and in [49], the authors show how sequential switching can be obtained with random connectivity. For decoding, hidden Markov models have been used for identifying clusters of neurons firing together in a trial-invariant sequence as a response to a taste stimulus in rodents, e.g. [50].

In firing-rate models, an influential model for sequential activation is the Laurent-Rabinovich model [11,21], where the connectivity between the neurons establishes a particular sequence of neuronal activations which is followed consistently across different trials. This model introduced the use of the Lotka-Volterra equations for sequential neuronal activations, which have been subsequently used successfully in areas as different as birdsong recognition [51,52], visual perception [53], handwriting recognition [54] and dendritic dynamics [55].

One interesting feature of the Lotka-Volterra equations as a modelling device of sequential activation of neurons is their remarkable robustness against unexpected input and noise and initial conditions different from zero, their trial-by-trial reproducible dynamics and the mathematical depth at which they have been studied previously [22,56].

Here, we incorporated both the sequential neuronal activations in the generative model, as introduced in [11,21], and the decoding mechanism to identify in an online fashion the odors with Bayesian inference. The SHS in the generative model grants the model with robustness against noise in the neuronal dynamics, which is further improved by the Bayesian inference which gives the model additional robustness against neuronal and sensory noise. The inclusion of clusters of KCs in our model, coupled with the ability of the Bayesian inference to reconcile conflicting data, gives the model a reliable and flexible mechanism as implemented by PN and KC activity.

### How many odors can the model recognize?

The insect brain is able to recognize many odors and even mixtures of odorants with different ratios [6,25] and for each one of these, a representation exists in the brain. In the proposed model, a high number of these representations can be encoded in a single connectivity matrix, like in the insect brain. Exactly how many sequences can be stored and recognized by the model will depend on a number of factors, most notably on the size of the KC population. Here, we discuss the capacity of the model in terms of the KC population’s size.

To explore the capacity of the model, we ran simulations with bigger population sizes to test how many sequences can be embedded while maintaining the model’s ability to accurately recognize them. We ran multiple simulations and found that the maximum number of sequences in a model grows faster than the size of the KC population and this growth is non-linear (e.g. polynomial). Importantly, we found that for KC numbers below 100, the system can store and accurately recognize only as many different odors as there are KCs (90). However, when going beyond 100 KCs, the number of odors that can be stored and recognized grows faster than the number of KCs. For example, for 500 KCs, 900 odors could be stored and recognized accurately (success rate > 95%). Simulations with more than 500 KCs are technically possible but we had to abandon these simulations because the computer run times became prohibitive (> 1 week on a modern desktop computer to assess the 500 KCs recognition performance). We are confident that the current Matlab implementation can be improved upon using an implementation using parallel computing (as used by the insect brain). It is an open question how many different odors could be recognized with 50,000 KCs (as in the locust). The proposed model would be an ideal tool to address this question, once the implementation is fast enough.

For future work, there are at least three ways in which one can improve the model’s capacity further, i.e. increase the number of recognizable odors while the number of KCs remains the same. Firstly, in this work we chose the sequences randomly, which means that some KCs can belong to many clusters. A more careful selection of the clusters in the sequences will most likely lead to an increase in the number of recognizable sequences. We expect that this is precisely what is happening in an insect brain, where KC connections are optimized, probably both during lifetime and by evolutionary processes. Secondly, the exact shape of the expected sequences (both at the PN and KC levels) plays an important role in the recognition. For our simulations, we used sequences similar to those in Fig 5B, where all KCs in a cluster rise at the same time and to the same maximum firing rate. A less stringent definition of a “sequence” will most likely lead to a much larger number of embeddable sequences. Thirdly, the exact weights of the connections between KCs may be improved further. While those used throughout this paper (see Fig 1) give accurate recognition results, a further optimization of the connectivity scheme could lead to a further improvement in model capacity.

## Methods

The model consists of two parts. The first one, called the generative model, is the part that sets the dynamics of neuron populations (both PNs and KCs). The second part is the Bayesian inference, which takes the output of the generative model (the PN population activity) and infers the hidden KC activity that is consistent with this input, identifying the presented odor, if it has been encountered before. For the generative model we used the Lotka-Volterra equations, to generate activity qualitatively similar to that observed in the mushroom body’s Kenyon cells (KC) and the projection neurons (PN) of the antennal lobe.

### Generative model

#### Lotka-Volterra equations for KC activity

The Lotka-Volterra equations were introduced as a model for sequential dynamics in olfaction in [11,21]. For a population of N neurons they are:

$${\dot{x}}_{i}=x_{i}\left(\sigma_{i}-\sum_{j=1}^{N}\rho_{ij}x_{j}\right)+\eta$$ 3

where *i* = 1,2,…, *N*, *x*_*i*_ is the state of the *i*-th neuron, *σ*_*i*_ is a parameter, *η* is noise and *ρ*_*ij*_ is the connectivity matrix between the neurons of the space. Under the condition *ρ*_*ii*_ = 1, for all *i* = 1,2,…, *N*, the system possesses a collection of equilibrium points of the form *Q*_*i*_ = (0,0,…,*σ*_*i*_,0,…,0), where the non-zero entry is at the *i*-th position. For our generative model, we dropped the dependence of *σ*_*i*_ and *ρ*_*ij*_ on the system’s input that was used in [11], making them instead a fixed parameter of the system.

A stable heteroclinic sequence (SHS) is a collection of equilibrium points and segments of trajectories that join them pairwise, i.e., a trajectory that goes from the first equilibrium point to the next, until the last one in a given sequence is reached. A stable heteroclinic channel (SHC) is a neighborhood around the SHS such that any trajectory that enters it will not leave it until the sequence ends at the last equilibrium point. A formal definition of these concepts can be found in [22].

Under the conditions in [22], the system in Eq 3 exhibits an SHS (and SHC). These conditions set the values of the connectivity matrix and of the constant terms *σ*_*i*_ in such a way that a neuron excites the next neuron in a sequence and highly inhibits all others. With this, the neurons are activated in a reproducible sequence.

For our model, we modified these conditions to enable us to have more than one component *x*_*i*_ be non-zero in an equilibrium point. Therefore, the equilibrium points in our system have the form *Q*_*i*_ = (0,0,…,*a*_*i*_,0,…,0,*a*_*i*_,…), where the non-zero entries are in positions *m*_*ij*_, with *j* = 1,…,*A*_*i*_, and *A*_*i*_ is the number of non-zero entries in this equilibrium point. The values of *a*_*i*_ are set by the parameters in Eq 3: for example, in the case where all the KCs in a cluster connect to each other with the same strength *ρ*, then all values *a*_*i*_ equal *σ*_*i*_ / *ρ*.

We now present conditions under which these points are equilibrium points of the system and an SHS arises. First, to guarantee that the points *Q*_*i*_ are equilibrium points, the following condition has to be met: For *x* = *Q*_*i*_,

$$\sigma_{i}-\sum_{j=1}^{A_{i}}\rho_{im_{ij}}x_{m_{ij}}=0$$ 4

For simplicity, we set *σ*_*i*_ = 1 for all *i* = 1,2,…, *N*, and to satisfy the condition in Eq 4, we set $\rho_{im_{ij}}={\left(A_{i}\right)}^{-1}$. The values for the connectivity matrix *ρ* were set as presented in ‘Model parameters used for simulations’ and Fig 1B.

We found that to preserve the stability of the SHS the connectivity matrix must obey Eq 4 and the within-cluster inhibition must be stronger than the inhibition from a cluster to the previous cluster in the sequence. Additionally, the number of embedded sequences (depending on the size of the population) must be low enough. We ensured that these conditions hold by using the reported values of *σ*_*i*_ and *ρ*_*ij*_.

#### Projection to PN space

The Lotka-Volterra equations in Eq 3 are used to model the behavior of the KC population in the insect brain. The neuronal states (firing rates) from these equations are then passed through an observation equation, which transforms these KC firing rates into PN firing rates, and is given by:

Y=ΘX+Γ 5

where *Y* is the *N*×1 vector of PN firing rates, *X* is the *M*×1 vector of KC firing rates from the Lotka-Volterra system, Θ is a *N* × *M* observation matrix, and Γ is Gaussian noise.

For every KC, we chose randomly a set of PNs that connect to it. The components of the observation matrix Θ are all ones and zeroes and implement the connections between KCs and PNs, i.e., if a KC connects to a PN, the corresponding component of Θ is 1, otherwise it is 0.

The generative model can be written in terms of prior and posterior distributions over the hidden states (KC firing rates) and observed states (PN firing rates) as follows:

$$\begin{array}{l} P(x_{1:T},y_{1:T})=P(x_{1})\prod_{t=2}^{T}P(x_{t}|x_{t-1})\prod_{t=1}^{T}P(y_{t}|x_{t})\\ \;P(x_{t}|x_{t-1})=N(F(x_{t-1},QI^{N\times N}))\\ \;P(y_{t}|x_{t})=N(g(x_{t}),RI^{M\times M}) \end{array}$$

where *x* is an N-dimensional vector of real numbers that represents the KC dynamics (firing rate) and *y* is an M-dimensional vector for the PN dynamics; *x*_1:*T*_ is a sequence of values for *x* obtained at times 1,2,…,*T*; *F*(*x*) is a discretized version of Eq 3 (see below), *g*(*x*) is the right-hand side of the observation equation (Eq 5) and *Q* and *R* are the state and observation noise precisions, respectively; **I** ^*N*×*N*^ and **I** ^*M*×*M*^ are identity matrices. N(·,·) is the Gaussian distribution.

To be able to work with Eqs 3 and 5, we must first discretize Eq 3 to obtain the form *x*_*t*+1_ = *F*(*x*_*t*_). We do this by integrating using the forward Euler method [58], transforming Eq 3 into:

$$x_{t+1}=x_{t}+\Delta tf(x_{t})$$ 6

where *f*(*x*_*t*_) is the right-hand side of Eq 3. Given the small time steps used during our simulations (Δ*t* ≤ 0.1), the forward Euler method provides a stable and precise solution to these differential equations. Similarly, the discretization of Eq 5 is:

$$y_{t}=g(x_{t})$$ 7

where *g*(*x*_*t*_) is the right-hand side of Eq 5. Note that *g*(*x*_*t*_) from Eq 5 contains a term of Gaussian noise, so it is not included in Eq 7.

### Bayesian inference

We implemented a Bayesian inference scheme that allows the system to identify a perceived stimulus. Similar setups, with Bayesian inference working on a generative model based on SHS, have proven fruitful in other applications [14,51,54]. In general, Bayesian inference observes variables whose states are known to be caused by other, hidden variables. Through the nonlinear equations of a generative model, which describe the way in which the hidden states cause the observed states, Bayesian inference balances the observed data with what it knows of the dynamics of the generative model in order to estimate the values of the hidden states that best fit the observed variables.

We made use of the unscented Kalman filter (UKF) as a Bayesian inference implementation [57]. The UKF works by comparing the data being observed at a given time step with a prediction made by the UKF in the previous step. These predictions are made using the generative model’s equations, so they follow its dynamics, and are done both for the observation and hidden variables. The difference between the observation and the prediction, called prediction error, is computed for both levels. These two prediction errors and the equations in the generative model are used to make the next-step prediction for both levels, such that the prediction error of one level can affect the prediction of the other. This back and forth exchange of information is what creates the extra connections in the model (i.e. the connections from PNs to KCs). At every time step in the process, these calculations and predictions are made and the expectations adjusted.

More specifically, the Bayesian inference continuously infers the states of the KCs from observed PN firing rates that were generated by Eq 5. We used the unscented Kalman filter (UKF) to estimate the states of the hidden variables *x*_*t*_ (the firing rates of the KCs) from the observed data *y*_*t*_ (the PN firing rates). At every time step *t*, the current estimates *x*_*t*_ and *y*_*t*_ (starting with some initial conditions for the first step, which we set to all zeroes or randomly), and a minimal set of points surrounding them, called sigma points, are propagated through the nonlinear equations of the generative system (Eq 6); assuming that the distribution of the hidden variables is Gaussian, a prediction for the mean and the variance of the hidden variables (KCs) and the observed variables (PNs) is calculated as a weighted sum of the propagated sigma points. In the next time step, these predictions are used to estimate the current states of the hidden variables with the update equation $x_{t}={\bar{x}}_{t}+K(y_{t}-{\bar{y}}_{t})$, where the Kalman gain *K* represents the precision expected from the data relative to the precision expected from the nonlinear dynamics of the system and is computed with the covariance matrices of the hidden and observed variables as calculated with the sigma points. In preparation for the next time step, a new prediction is calculated using the current estimation and the process is repeated.

The filter takes as input the PN activity and as priors an initial condition of the KC population and the covariance matrix for the noise vectors (*H*)_*i*_ = *η*_*i*_ from Eq 3 and Γ from Eq 5. We set the initial state of the KCs to zeroes (or randomly, for testing for robustness) and the noise covariance matrices (priors) are unit matrices multiplied by two different constants (*Q* for the hidden states, *R* for the observed states). These constants, called precisions, determine the relative importance given either to the observations (PN readings) or to the internal dynamics of the KCs. For all our simulations, we used values of *Q* = 0.1 and *R* = 0.001. These values were chosen because they provide the best balance between following the observed PN data and following the expected KC dynamics.

Given that Bayesian inference does not require the entire data set, but only the data for the given step and the prediction from the previous one, this process can be done online, that is, it can be done as data acquisition is happening, without having to wait for the entire process to be over. This characteristic makes the Bayesian inference a plausible mechanism through which the brain can decode the information encoded in the PN responses.

### Simulation setup

In this section, we present an overview of the steps necessary to implement our model. To complement this, source code for the implementation, as well as examples, can be found at [https://github.com/dcuevasr/Olfaction/]. The simulations are divided in two steps: firstly, data generation; secondly, inversion.

For the data generation phase, the following steps were followed: (1) Set-up the parameters of the system, e.g. population sizes. (2) Select the clusters that will form the sequences. We did this randomly, minimizing repetition of neurons in different sequences. (3) Create the connectivity matrix, embedding all the desired sequences in it. (4) Set-up the initial conditions for all the neurons. This step determines which sequence will be generated: to generate data with a sequence, the initial conditions must be around the first equilibrium point of that sequence, i.e. the first cluster of the sequence must be activated. (5) Integrate Eq 3 to obtain KC activity. (6) Randomly generate an observation matrix and generate PN activity using Eq 5.

Of special note are the algorithms for creating the clusters and sequences, and for generating the connectivity matrix. These can be seen in the files cgenerate_clusters.m and cget_rho.m, respectively. Additionally, the generation of the observation matrix can be seen in the file cgenerate_neurons.m.

For the inversion phase, the steps are the following: (1) Set the precision parameters. (2) Set the initial conditions, which were typically set to zeroes. (3) Call the UKF using the PN data generated previously as input. The implementation of the UKF is contained in the file UKF.m.

### Raster plots

To generate the illustrative raster plots shown in Fig 3, we adapted our generative model to generate spikes while keeping its fundamental structure. We created a hierarchical three-level model, where the third (top) level employs the proposed cluster-encoding Lotka-Volterra equations (see Fig 1). These are output to the second level, representing the KCs (Fig 3A, right panel), which are modeled using FitzHugh-Nagumo equations to generate spikes [59]. The input from the third to the second level raises the membrane potential in the FitzHugh-Nagumo equations to the threshold of spiking. Spikes are generated by adding noise to pass the firing threshold. For simplicity, we used Gaussian noise.

The states of the second level (representing the membrane potential of the KCs) are the input to the first (lowest) level, which represents the PNs, also modeled with the FitzHugh-Nagumo equations (see Fig 3A, left panel) in the same way: Only when the input from the second level takes the membrane potential close to threshold, spikes are occasionally generated both by Gaussian noise and by the spikes of the second level.

The connections between the PNs and the KCs in this model are made in the same way as in our model: by a random 1 to 20 projection from KCs to PNs with equal weights.

This model was only used to generate the data to create the plots in Fig 3. Because of this, we did not apply Bayesian inference to it.

For a full description of the three-level hierarchical model see S1 Text.

### Decision making: Choice and reaction time

When making decisions and to generate reaction times, we compare the KC response obtained by the Bayesian inference with the expected KC response (used to generate the data). In particular, we compute the Euclidean distance between the two trajectories in the firing rate phase space of the KCs and define recognition of an odor as correct, if the Euclidean distance drops below a threshold anytime during the trial. We further defined reaction time as the time at which the threshold is crossed.

We set a threshold of 0.1 for our simulations, which is 0.1 times the maximum value of the firing rate of a KC. We found that once this threshold is crossed, the two trajectories (inferred and the data) do not drift apart for the rest of the response, making this threshold a good assessment of whether the inference is choosing the correct representation.

In the section ‘Intelligent coincidence detector’, we make use of this criterion to compare the performances of our model and the single-neuron modification in order to quantify the effect of the lateral inhibition received by KCs from those KCs with which it shares a cluster. For each model, we ran a hundred trials each for all combinations of the number of extra-noisy PNs (between one and twenty) and extra noise added to these PNs (with SNRs of 2, 1.25 and 1). For each of these trials, we considered the odor to be properly identified if the Euclidean distance between the inferred and expected responses was lower than the 0.1 threshold.

In Fig 6, the variable *Recognition*, which goes from -1 for the incorrect representation to 1 for the correct (expected) one, was calculated using the following formula:

$$R=\frac{D_{c}-D_{i}}{D_{ri}}$$ 8

where *R* is the recognition, *D*_*c*_ is the distance between the observed KC activity and the correct (expected) one; *D*_*i*_ is the distance between the observed activity and the incorrect one (the other sequence in the system) and *D*_*ri*_ is the distance between the two sequences embedded in the connectivity matrix.

### Full stimulus data

We created PN activity which presents the three phases of the response to an odor (see Fig 7). To do this, we generated the response in the KCs for one sequence for the first phase, then repeated the last point in the sequence for a certain time to generate the response in the second phase; finally, we added the response of a second sequence (embedded in the same connectivity matrix) at the end for the third phase (Fig 7B). We created the PN response with a 1:20 projection (i.e. each KC is connected to 20 PNs), as used in the other simulations. Using this full response PN activity as input to the Bayesian inference, we measured the inferred KC responses.

## Supporting Information

S1 Text Hierarchical spiking model. Description of a hierarchical spiking model used in the generation of Fig 4.
(PDF)
