# Supplementary material for: Modelling Odor Decoding in the Antennal Lobe by Combining Sequential Firing Rate Models with Bayesian Inference
Source: PLoS Comput Biol. 2015 Oct 9;11(10):e1004528. doi: 10.1371/journal.pcbi.1004528 (PMC4599861; doi:10.1371/journal.pcbi.1004528)
Supplement: S1 Text — Description of a hierarchical spiking model used in the generation of Fig 4. (PDF) [file pcbi.1004528.s001.pdf]

Supplementary Information

Cuevas Rivera, Dario; Bitzer, Sebastian; Kiebel, Stefan

1 Generative model

Here, we describe a three-level hierarchical generative model that was used in the generation of Figure 3 and related text.
This model, which was not inverted for the current work, contains three levels. The first level (bottom) represents the membrane potential of a population of PNs. The second level (middle) represents the membrane potential of the KCs. The third level (top) is an abstract level, in the sense that it does not represent any physical part of the antennal lobe or mushroom body. This level creates the sequential activity observed in the antennal lobe and the mushroom body and is based on the Lotka-Volterra equations, as were used for our proposed model in the main text (see the Methods section). In this document, we will describe in full detail these three levels and how the plots in Figure 3 were generated with it.
We will describe the model for a population of N KCs and M PNs; the number of connections between KCs and PNs will be as in the main text: $C = 20$, meaning 20 KCs connect to each PNs.

1.1 Top level

We will begin by describing the top level. This level is based on the Lotka-Volterra equations, which are as follows:

$$\dot{x}_i^{(3)} = x_i^{(3)} \left(\sigma_i + \sum_{j=1}^{N} \rho_{ij} x_j^{(3)} \right) + \eta_i^{(3)}, \qquad i = 1, 2, ..., N \tag{1}$$

This level behaves exactly as the KC level of the generative model of the proposed model in the main text. Thus, we choose $\sigma_i = 1, \forall i$ and the connectivity matrix ρ_{ij} contains all the sequences stored in the system; the values of ρ_{ij} were set as described in Figure 1 of the main text. The solution to these equations follows the sequential dynamics that will be at the heart of the behavior of this three-level hierarchical model.

1.2 Middle level: KCs

To model the membrane potential of the KCs, we make use of the FitzHugh-Nagumo (FHN) model **?**. We use this model for its simplicity in generating action potentials. The equations of the FHN model are the following:

$$\dot{x}_i^{(2)} = x_i^{(2)} - \frac{(x_i^{(2)})^3}{3} - w_i^{(2)} + I_{\text{ext}}^{(2)} \tag{2}$$

$$\tau \dot{w}_i^{(2)} = x_i^{(2)} + a - b w_i^{(2)} \tag{3}$$

where $x_i^{(2)}$ is the membrane potential of neuron i and $w^{(2)}$ is a recovery variable; The values for the parameters a, b and τ were chosen as in **?**, and are $a = 0.7$, $b = 0.8$, $\tau = 12.5$. We used as initial conditions $v(t = 0) = w(t = 0) = -1.2$. This parameter regime ensures that no action potentials are generated when the input $I_{\text{ext}}^{(2)}$ is zero.
The input to this middle level depends linearly on the output of the top level and has the same form for all KCs, i.e.

$$I_{\text{ext}}^{(2)} = \gamma x^{(3)} + \Gamma_i^{(2)} \tag{4}$$

where γ is a fixed parameter and Γ_i is Gaussian noise. For our simulations, we used $\gamma = 0.3$; this value was chosen such that when $x_i^{(3)}$ was at its maximum value, the membrane potential of $x_i^{(2)}$ is right under the firing threshold. Then, during the time where $x_i^{(3)}$ is at its maximum, spikes can be observed in the i-th KC depending on the noise Γ_i. This means that while the period during which action potentials can be observed is fixed, the exact number and timing are random.
The firing rate of a KC can be controlled in two ways. The first option is to change the value of γ to take the membrane potential closer or further away from the firing threshold. The second option is to change the variance of the noise Γ_i. For the illustrations in Figure 3 of the main text, we used $\Gamma_i = \mathcal{N}(0, 0.35)$.

1.3 Bottom level: PNs

The bottom level represents the membrane potentials of the PNs in our model. We will again make use of the FitzHugh-Nagumo model for these neurons. The equations are then as follows:

$$\dot{x}_i^{(1)} = x_i^{(1)} - \frac{(x_i^{(1)})^3}{3} - w_i^{(1)} + I_{\text{ext}}^{(1)} \tag{5}$$

$$\tau \dot{w}_i^{(1)} = x_i^{(1)} + a - b w_i^{(1)} \tag{6}$$

with the same parameters as with the middle level. In similar fashion,

$$I_{\text{ext}}^{(1)} = 2\Theta(1.2 + x^{(2)})/C + \Gamma_i^{(1)} \tag{7}$$

where Θ is the projection matrix (see eq. 5 in main text) and C is the number of KCs connecting to a PN; $\Gamma_i^{(1)} = \mathcal{N}(0, 0.6)$. As with the second level, this ensures that when the KCs that connect to a PN are near the firing threshold, the PN is there too. One difference between the ways in which $I_{\text{ext}}^{(2)}$ and $I_{\text{ext}}^{(1)}$ affect their corresponding neurons is that $I_{\text{ext}}^{(2)}$ does not create spikes on its own in the middle level, while $I_{\text{ext}}^{(1)}$ is capable of doing so because $x_i^{(2)}$ itself can contain spikes which would then cause spikes on the PNs that it connects to. In other words, a spike in a KC of the middle level is very likely to cause a spike in the PNs that it connects to in the bottom level and spikes can be observed in the bottom level even in the absence of noise.

2 Raster plots

The raster plots in Figure 3 were generated using the membrane potentials $x_i^{(2)}$ and $x_i^{(1)}$ for KCs and PNs, respectively. Any time the membrane potential of KC or PN became positive, a spike is said to be observed. Using this simple criterion, the raster plots were created.
